# Group 2 innate lymphoid cells contribute to IL-33-mediated alleviation of cardiac fibrosis

**DOI:** 10.7150/thno.51648

**Published:** 2021-01-01

**Authors:** Wei-Yu Chen, Yi-Hsiu Wu, Tzu-Hsien Tsai, Ru-Fang Li, Alan Chuan-Ying Lai, Lung-Chih Li, Jenq-Lin Yang, Ya-Jen Chang

**Affiliations:** 1 Institute for Translational Research in Biomedicine, Kaohsiung Chang Gung Memorial Hospital, Kaohsiung, Taiwan.; 2 Institute of Biomedical Sciences, Academia Sinica, Taipei, Taiwan.; 3 Division of Cardiology, Department of Internal Medicine, Kaohsiung Chang Gung Memorial Hospital and Chang Gung University College of Medicine, Kaohsiung City, Taiwan.; 4 Division of Cardiology, Department of Internal Medicine, Kaohsiung Medical University Hospital, Kaohsiung Medical University, Kaohsiung City, Taiwan.; 5 Division of Nephrology, Department of Internal Medicine, Kaohsiung Chang Gung Memorial Hospital and Chang Gung University College of Medicine, Kaohsiung City, Taiwan.

**Keywords:** interleukin-33, cardiac fibrosis, ILC2, fibroblast activation, myocardial injury

## Abstract

**Rationale:** The major cause of heart failure is myocardium death consequent to detrimental cardiac remodeling and fibrosis following myocardial infarction. The cardiac protective cytokine interleukin (IL)-33, which signals by ST2 receptor binding, is associated with group 2 innate lymphoid cell (ILC2) activation and regulates tissue homeostasis and repair following tissue injury in various tissues. However, the distribution and role of IL-33-responsive ILC2s in cardiac fibrosis remain unclear. In this study, we elucidated the roles of IL-33-responsive cardiac-resident ILC2s and IL-33-mediated immunomodulatory functions in cardiac fibrosis.

**Methods:** We examined the distribution of cardiac ILC2s by using flow cytometry. The roles of IL-33-mediated ILC2 expansion in cardiac fibrosis was evaluated in the mouse model of catecholamine-induced cardiac fibrosis. ILC-deficient *Rag2^‒/‒^IL2Rγc^‒/‒^* mice were implemented to determine the contribution of endogenous ILC in the progression of cardiac fibrosis. Histopathological assessments, speckle tracking echocardiography, and transcriptome profile analysis were performed to determine the effects of IL-33-mediated cardiac protective functions.

**Results:** We identified the resident cardiac ILC2s, which share similar cell surface marker and transcriptional factor expression characteristics as peripheral blood and lung tissue ILC2s. IL-33 treatment induced ILC2 expansion via ST2. *In vivo*, ILC-deficient *Rag2^‒/‒^IL2Rγc^‒/‒^* mice developed exacerbated cardiac fibrosis following catecholamine-induced stress cardiac injury. IL-33 treatment expanded cardiac ILC2s and revealed protective effects against cardiac tissue damage with reduced cardiomyocyte death, immune cell infiltration, tissue fibrosis, and improved myocardial function. Transcriptome analysis revealed that IL-33 attenuated extracellular matrix synthesis- and fibroblast activation-associated gene expressions. *IL13*-knockout or epidermal growth factor receptor (EGFR) inhibition abolished IL-33-mediated cardiac protective function, confirming IL-13 and EGFR signaling as crucial for IL-33-mediated cardioprotective responses. Moreover, ILC2-produced BMP-7 served as a novel anti-fibrotic factor to inhibit TGF-β1-induced cardiac fibroblast activation.

**Conclusion:** Our findings indicate the presence of IL-33-responsive ILC2s in cardiac tissue and that IL-33-mediated ILC2 expansion affords optimal cardioprotective function via ILC2-derived factors. IL-33-mediated immunomodulation is thus a promising strategy to promote tissue repair and alleviate cardiac fibrosis following acute cardiac injury.

## Introduction

Cardiac fibrosis, a condition attributed to the excessive deposition of extracellular matrix (ECM) components produced by activated fibroblasts and/or myofibroblasts, constitutes a common feature associated with nearly all forms of heart disease 1, 2. Extensive cardiac fibrosis results in cardiac structural changes and stiffening, conductivity problems, and reduced oxygen diffusion, leading to diminished ventricular function and arrhythmia 3.

Interleukin (IL)-33, an IL-1 family cytokine that functions as a tissue-derived nuclear alarmin 4, is constitutively expressed in epithelial barrier tissues and endothelial cell barriers 5, 6. The IL-33 receptor complex consists of the ST2 subunit, encoded by the *IL1RL1* gene 5, 7, encompassing two major transcription variants: the full-length transmembrane form (ST2L) and the soluble form (sST2) 8. sST2 lacks the transmembrane domain and binds to IL-33 as a decoy receptor 9. Endothelial cells from blood vessels exhibit high IL-33 levels in human 10 but not mouse,^6^ whereas murine IL-33 can be induced in endothelium in inflamed tissues 6. Apoptotic cells inactivate endogenous IL-33 by caspases to avoid alerting the immune system 11, 12. Full length bioactive IL-33 is released to the extracellular space upon tissue damage and cell necrosis (or cell stress) following allergen exposure or infection 13-15. Secreted IL-33 triggers the immune system by activating various types of immune cells including mast, T helper 2, and group 2 innate lymphoid (ILC2) cells, which secrete large amounts of IL-5, IL-13, and amphiregulin (Areg) 15-17. ILC2s have been identified in mucosal organs including the lung 18 and may play protective roles in atherosclerosis in mice 19, along with regulation of tissue homeostasis, repair, and inflammatory response upon infection or tissue damage 20. Recently, a rare cell population exhibiting ILC2-like characteristics was identified in mouse heart using single cell sequencing and flow cytometry 21, 22. However, the characteristics and pathophysiological roles of cardiac-resident ILC2s and their effector functions in the heart upon IL-33 stimulation remain to be elucidated.

We and others found that IL-33 exerts cardioprotective functions in animal models of cardiac pressure overload induced by transverse aortic constriction surgery 23, 24 and myocardial infarction 25, 26. Using tissue specific conditional knockout mice, we confirmed that endothelial-derived IL-33 mediated the anti-hypertrophic responses in ST2-expressing cardiomyocytes 24. Moreover, we found that IL-33 elicits selective inflammatory responses through IL-13 and TGF-β1 24. However, although IL-33 exerts cardioprotective function following cardiac injury and has been linked to activation of type 2 immune responses via ILC2s, whether the IL-33-responsive ILC2s directly contribute to IL-33-mediated immunomodulatory function and regulate tissue repair and fibrosis in the injured myocardium remain largely unknown.

In this study, we identified and characterized the IL-33-responsive cardiac ILC2 population resident in the mouse heart. IL-33 treatment expanded the cardiac ILC2 population via the ST2 receptor and elicited protective effects against catecholamine-induced cardiac fibrosis with reduced cardiomyocyte death, immune cell infiltration, cardiac fibroblast (CF) activation, fibrotic gene expressions, and improved myocardial function. Transcriptome analysis revealed that IL-33 attenuated ECM synthesis- and fibroblast activation-associated gene expression. We demonstrated that ILC2-derived factors including IL-13, Areg, and BMP-7 concurrently contribute to IL-33-mediated anti-fibrotic responses following acute cardiac injury. Overall, our results revealed that IL-33-responsive ILC2s contribute to IL-33-mediated cardiac protective function in alleviation of cardiac fibrosis.

## Materials and Methods

### Animals

Mice were maintained in the AAALAC-certified specific pathogen-free animal facility of Kaohsiung Chang Gung Memorial Hospital (KCGMH) with controlled temperature and light cycles (24 ºC and 12/12 h light/dark cycle) with *ad libitum* access to regular food and water. All experimental animal protocols were approved by the KCGMH IACUC committee (2015122507 and 2018122122). Both male and female mice aged 6-14 weeks were used for all experiments. Wild-type C57BL/6JNarl and BALB/cByJNarl mice were purchased from National Laboratory of Animal Center (NLAC), Taiwan. B6.*ST2*^‒/‒^, B6.*ST2^‒/‒^ IL33^‒/‒^*, and B6.*IL33^‒/‒^* mice were generated as described in our previous study 27 and maintained on a C57BL/6JNarl background.* Il1rl1*^-/-^ (ST2 knockout) 28 and *Il13*^-/-^ 29 mice were kindly provided by Dr. Andrew McKenzie (MRC Laboratory of Molecular Biology, Cambridge, UK). BALB/c.*Rag2^‒/‒^* (Taconic, Model 601), and BALB/c.*Rag2^‒/‒^IL2Rγc^‒/‒^* (Jackson Laboratory, 014593) mice were maintained on a BALB/c background.

### Flow cytometry

Murine blood was harvested through the retro-orbital route and transferred into heparin-containing canonical tubes. Blood leukocytes were purified by using Histopaque 1077 and 1119 (Sigma) following the manufacturer's instructions. For isolation of cells from the lung and heart, mice were anesthetized with Isoflurane (3%) and the heart and lung tissues were dissected and then flushed by phosphate-buffered saline (PBS) through the ventricles to remove excessive blood content. Lung tissues were minced by razor and then digested in DMEM (3 mL) containing collagenase IV (1.6 mg/mL) and DNase I (0.1 mg/mL) (37 °C, 45 min), using an 18-gauge needle to shear tissue during digestion. Homogenates were filtered using 75-μm mesh and then centrifuged (400 × g, 5 min). Pellets were incubated in RBC lysis buffer (Gibco or Biolegend) (4 °C, 5 min). The isolated cells were neutralized with PBS. Similar procedure was performed for isolation of cardiac cells, except the digestion buffer comprised DMEM (2 mL) containing collagenase B (1.0 mg/mL) and DNase I (0.1 mg/mL) (37 °C, 30 min).

After homogenization, the cells were treated with 2.4G2 conditioned medium or TruStain FcX (Biolegend 101320) (4 °C, 10 min) to block Fc gamma receptors on leukocytes. Fixable Viability Dye (Thermo Fisher) and fluorescence-conjugated antibodies were then applied (4 °C, 30 min). To stain transcription factors, cells were initially permeabilized using the Foxp3 Transcription Factor Staining Buffer Set (Thermo Fisher). Then, the 2% FCS/PBS-washed cell suspensions were analyzed using the fluorescence-activated cell sorter (FACS) LSRII (BD) or by Gallios (Beckman Coulter). Flow diagrams were generated using FlowJo v.10.1 (TreeStar). Details of antibodies used for flow cytometry are listed in **Table S1**.

### Expression and purification of recombinant mouse IL-33

Mouse IL-33 cDNA (Ser^109^-Ile^266^) containing a 6X Histidine tags was synthesized with E. Coli favorable codons (Genewise Inc.). The synthesized cDNA was then cloned into pET15b expression vector (Novagen Inc., Madison, WI, USA). The protein was expressed mainly in soluble fraction. Recombinant mouse IL-33 protein was purified by using Ni-NTA affinity chromatography (17-5248-02, GE Healthcare Life Sciences) using BioLogic LP System (Bio-Rad). Following the protein purification, the protein solution was applied through the Endotoxin Removal Spin Columns (#88275, ThermoFisher Scientific) to remove endotoxin. The endotoxin levels in the purified protein was analyzed and confirmed with <0.01 EU per 1 μg of the protein by the LAL method (#88282, ThermoFisher Scientific). The purity of the recombinant proteins was evaluated by Coomassie blue staining analysis and Western blot, which was performed with anti-His antibodies.

### Isoproterenol (ISO)-induced cardiac injury

For acute isoproterenol-induced cardiac injury, male or female wild-type BALB/cByJ mice (age between 8-12 week-old) were randomly subjected into groups and were subcutaneously injected with normal saline as a control or ISO (Santa Cruz, sc-294398) for three consecutive days (60 mg kg^-1^ d^-1^) to induce acute cardiac injury as previously described 30 with modifications. For IL-33 post-treatment, recombinant mouse IL-33 (mIL-33, 0.5 µg per mouse) was intraperitoneally administrated on day 3, 5, and 7 following the last ISO injection and the hearts and sera were collected on day 10 for histology, mRNA, and serum cytokine analyses. For IL-33 pre-treatment, mice were administered mIL-33 prior to ISO challenges and the heart tissues were collected on day 10 for histological assessments. For IL-33 co-treatment, mice received mIL-33 with ISO challenges and cardiac tissues were collected for flow cytometry analysis on day 4 after the last ISO injection. To examine the effect of epidermal growth factor receptor (EGFR) inhibitors, 10 mg/kg geftinib (Selleckchem, #S1025) or 10 mg/kg afatinib (#S7810) were dissolved in dimethylsulfoxide and intraperitoneally injected with IL-33 (0.5 µg per mouse) on day 3, 5, and 7 post ISO treatment. The same experimental protocol for ISO treatment and IL-33 treatment was performed in *IL13^‒/‒^* mice (male or female mice between 8-12 week-old).

For ISO-induced cardiac fibrosis in BALB/c.*Rag2^‒/‒^* (Taconic, Model 601), and BALB/c.*Rag2^‒/‒^IL2Rγc^‒/‒^* mice, the mice (male or female between 8-12 week-old) were randomly subjected to subcutaneous injection with Saline or ISO (30mg/kg per day for 3 days). The hearts were isolated for histological assessments on day 4 post last ISO treatment. For evaluation of the effect of IL-33 on cardiac fibrosis in BALB/c.*Rag2^‒/‒^IL2Rγc^‒/‒^*, the mice (male or female between 8-12 week-old) were randomly subjected to subcutaneous injection with Saline or ISO (30mg/kg per day for 3 days). Recombinant mouse IL-33 (mIL-33, 0.5 µg per mouse) was intraperitoneally administrated on day 3, 5, and 7 following the last ISO injection and the hearts and sera were collected on day 10 for histology, mRNA, and serum cytokine analyses.

### Histopathological assessment, immunohistochemical and immunofluorescence staining

Mouse heart tissues were fixed with 4% paraformaldehyde, embedded in paraffin, sectioned, and stained using standard immunohistochemistry and fluorescence microscopy methods as previously described 27. An additional antigen retrieval step was applied in all experiments by heating samples in a Tris-based buffer (pH 9.0) to 95 °C for 20 min. Primary antibodies are listed in **Table S1**. Alexa Fluor-conjugated secondary antibodies were purchased from ThermoFisher. Slides were incubated with 1% Sudan black (Sigma-Aldrich) in 75% ethanol at room temperature for 20 min to reduce tissue auto-fluorescence before mounting. Confocal images were captured using an Olympus FLUOVIEW FV10i confocal microscope. For fibrosis area quantification, paraffin sections were stained with hematoxylin and eosin or Picrosirius red stain kit (Abcam, #150681) per manufacturer instruction. Bright-field images were scanned using 3D Histech Pannoramic MIDI (3DHISTECH) under 20 X magnification. Eight random fields of the left ventricular regions were captured using the Pannoramic viewer (3DHISTECH) and the red-stained area quantified using ImageJ software (NIH). Red-stained fibrotic areas and non-stained myocyte areas from each section were determined using color-based thresholding. Total fibrosis area percentage was calculated as the summed red-stained areas divided by the total cardiac area. All histological assessments were performed by two technicians blinded to treatment groups.

### ILC2 isolation and conditioned medium collection

*Rag2^‒/‒^* mice were intranasally treated with IL-33 (1 μg) and sacrificed five days later. Lung ILC2 cells (Lin^-^ST2^+^ subset) were sorted using FACSAria III and cultivated in RPMI-1640 supplemented with IL-2, IL-7, and IL-33 (all at 10 ng/mL). Cells were maintained for 72 h in RPMI-1640 containing only IL-2 and IL-7 (10 ng/mL) prior to each experiment. To collect IL-33-activated ILC2 conditioned medium (ILC2-CM), the cells were treated with IL-2, IL-7, and IL-33 (10 ng/mL) for 48 h. CMs were collected for ELISA and CF activation experiments. For the adoptive transfer experiments, *Rag2^‒/‒^IL2Rγc^‒/‒^*mice were challenged with ISO for three doses (30mg/kg/d). Then, ILC2 cells (5 × 10^6^ cells) were injected via tail vein following last dose of ISO. The hearts were harvested for histological assessments on day 4 post last ISO injection.

### Transcriptome analysis by microarray

The mouse heart or cardiac fibroblast RNA samples were subjected to gene expression profiling using Affymetrix MTA 1.0 microarray chips. RNA samples were prepared using the WT PLUS Reagent kit (Affymetrix) followed by microarray hybridization. The raw data were first subjected to quality control examination per manufacturer guidelines. Chips passing the quality control criteria were then analyzed using Partek Genomics Suite (Missouri, USA). CEL files were further analyzed using the Affymetrix Transcriptome Analysis Console. Differentially expressed gene clustering, functional annotation, and gene ontology analyses were performed by using STRING (https://string-db.org).

### Speckle tracking echocardiography

Echocardiography was performed in mice anesthetized with 1.5% isoflurane, using a 40 MHz linear-array transducer in conjunction with a digital ultrasound system (Vevo 2100 Imaging System, VisualSonics). The average of three consecutive cardiac cycles was used. Left ventricular fractional shortening was calculated as a percentage as follows: (EDD - ESD)/EDD × 100, where EDD is end-diastolic dimension and ESD is end-systolic dimension. The observer was blind to the treatments. Cardiac functions including heart rate, left ventricular end-diastolic diameter, left ventricular end-systolic diameter, posterior wall thickness in diastole, interventricular septum thickness in diastole, fractional shorting, and ejection fraction were assessed. To minimize the confounding influence of different heart rates on aortic pressure gradient and left ventricular function, the flow of isoflurane (inhalational) was adjusted to anesthetize the mice while maintaining their heart rates at 450-550 beats per minute. During speckle tracking echocardiography analysis, the left ventricle was automatically divided into six segments. Strain curves were made available for each analyzed segment. Data for the peak longitudinal and radial segmental strain and strain rate, and the time to peak strain (time from the onset of the QRS complex to peak strain value) were analyzed according to instrumental instruction. Echocardiography was performed by an experienced cardiologist blinded to the treatment group. M-mode standard two-dimensional (2D) long axis echocardiographic ex-amination was conducted, and the fractional shortening (FS), ejection fraction (EF) were calculated by Vevo 2100 software. For measuring the pulsed wave Doppler spectral waveforms, we measured the peak early‐ and late‐diastolic transmitral velocities (E and A waves) to obtain the E/A ratio.

### Primary cardiac fibroblast (CF) isolation and culture

Primary CFs were isolated from 2-day old neonatal C57BL/6JNarl mice (NLAC). Briefly, neonatal mouse hearts were isolated and quickly placed in a cell culture dish containing 75% ethanol solution, then transferred to ice-cold PBS, minced into small pieces using curved scissors, and transferred into 50 mL of Trypsin-EDTA solution (0.05%, Gibco, #15400-054) with 100 U/mL collagenase II (Worthington, #LS004177). The tissues were incubated (37 ºC, 30 min), gently agitating 2 to 3 times. The tube was then centrifuged (5 min, 2000 rpm) and the supernatant was removed. The cells were re-suspended by adding CF culture medium (DMEM/F12; Gibco #11330-032) (10 mL) containing L-ascorbic acid (Sigma, #A4544) (100 μM), 10% fetal bovine serum (Gibco #10437-028), and 1 X penicillin/streptomycin solutions (Gibco, #15140122)), gently mixed by repeatedly pipetting up and down 20-30 times, plated into 6 well cell culture dishes (Corning, Costar #3516) pre-coated with gelatin (1% in PBS) for 1 day prior to the experiment, and incubated at 37 ºC in a cell culture incubator containing 5% CO_2_ for 4 h to allow cell attachment. Non-attached cells were removed, and the dishes were washed 2 to 3 times using 1X PBS. The cells were cultured in CF culture medium and the medium was changed every 2 days and cultured for 7 days before further subculture.

To evaluate IL-33 or ILC2-CM effects on CF activation, primary CFs (1 × 10^5^ cells) were treated with Saline control, mIL-33 (Biolegend, 30 ng/mL), ILC2-CM (1:100), hTGF-β1 (Peprotech, 30 ng/mL), TGF-β1+IL-33, or TGF-β1+ILC2-CM for 48 h. The cells were harvested, and the mRNA of the samples was isolated for qRT-PCR analysis of the gene expression fibroblast activation markers (*Postn* and *Tnc*).

### Quantitative real-time polymerase chain reaction (qRT-PCR)

Total RNA from the heart tissues or cells was isolated using TRIzol reagent (Invitrogen). cDNA was synthesized from 1 µg of total RNA and random hexamers using the High-Capacity RNA-to-cDNA^TM^ Kit (ABI (4387406). qRT-PCR was performed using an ABI 7300 Real-Time PCR system (Applied Biosystems) with the Quantinova SYBR Green PCR Kit (Qiagen, 208054). The primer sequences used are listed in **Table S2.** The relative quantity of the target transcript was normalized to endogenous *Hprt* transcript levels using the 2^-∆∆Ct^ method. Each sample was run in triplicate and data are presented as the means ± SD.

### ELISA

Supernatants of sorted ILC2s were harvested after 48 h of culture with or without IL-33. The cytokine levels in the supernatants (IL-5, IL-13, IL-10, BMP-7, Areg, and HB-EGF) and mouse serum (BMP-7, Areg, and HB-EGF) were analyzed by ELISA according to manufacturer instruction. ELISA kits used are listed in **Table S3**. Serum cytokine concentrations (IL-5, IL-13, IL-10, and G-CSF) were measured using an antibody-coated microsphere-based multiplex cytokine immunoassay with 25 µL of serum samples (MILLIPLEX MAP Mouse Cytokine/Chemokine Magnetic Bead Panel, MERCK Millipore Corporation).

### Statistical analyses

Statistical significance between two groups was analyzed using the nonparametric Mann-Whitney test. The significance of differences among the groups was evaluated using one-way analysis of variance, followed by the Bonferroni multiple comparison post-hoc test. Statistical analysis was performed using Prism 6 statistical software (GraphPad). All data are reported as the means ± SD. Statistical significance was set at *P*<0.05.

### Data availability

All data supporting this study can be found within the paper and its Supporting Information files. Additional data supporting the findings of this study are available from the corresponding authors (W-Y. C and Y-J. C.) upon reasonable request.

## Results

### Characterization of the resident cardiac ILC2 population

**Figure 1:**
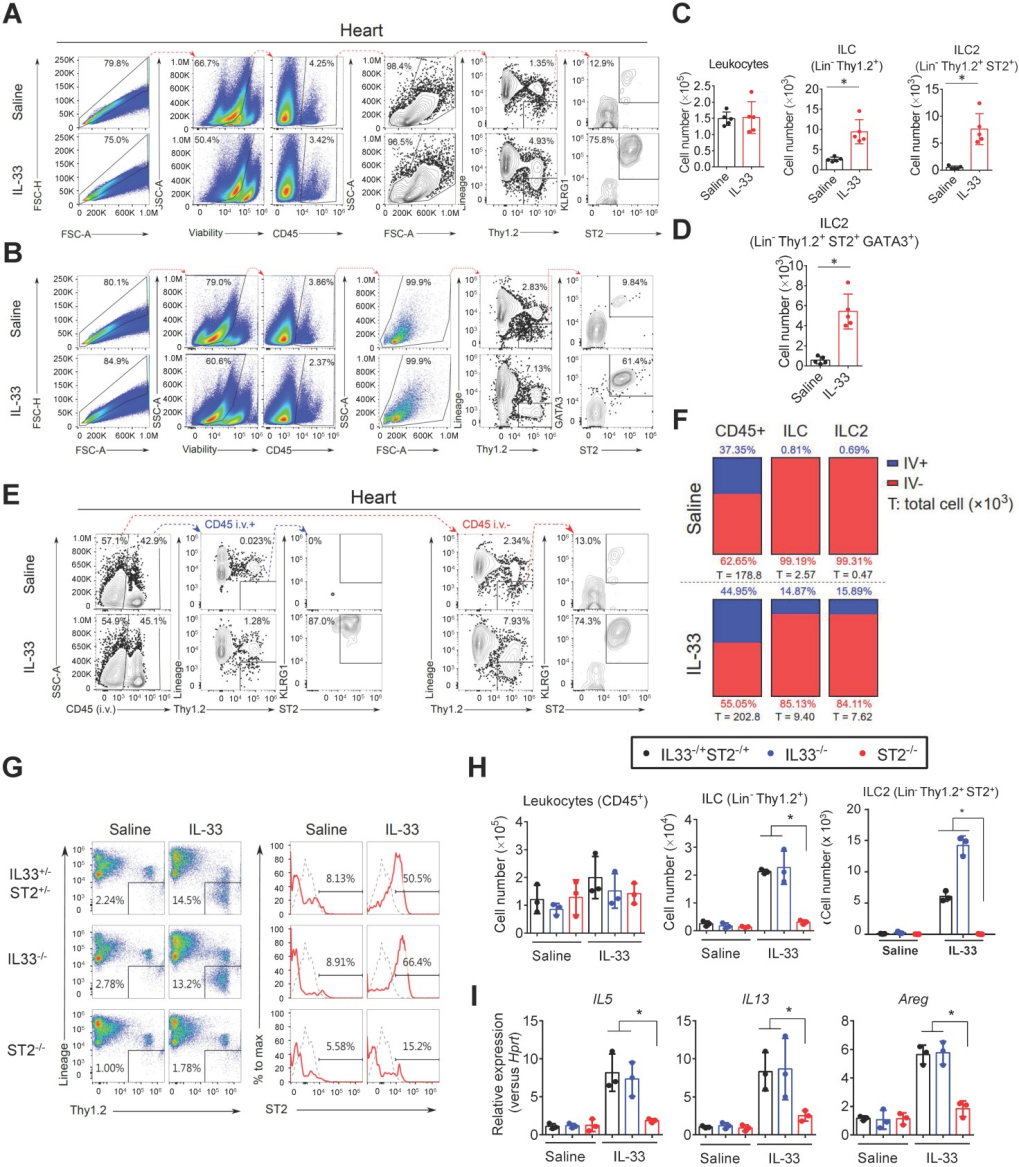
** Identification and characterization of the cardiac resident ILC2 population.** Wild-type C57BL/6J mice were intraperitoneally injected with Saline or IL-33 (2 μg/mouse for 5 consecutive days). The heart tissues were collected for flow cytometry analyses of ILC2 population surface markers and transcription factors. **(A)** Representative plots and gating strategy for ILC (CD45^+^Lin^-^Thy1.2^+^) and ILC2 population (CD45^+^Lin^-^Thy1.2^+^ST2^+^) identification in mouse heart cells. **(B)** Representative plots for characterization of GATA3 expression in mouse cardiac ILC2s. Lineage markers (CD3, CD19, FcεRI, CD11c, CD11b, F4/80, and CD49b) were used in the flow cytometry. (**C**) Total number of CD45^+^ leukocytes, ILC, ST2^+^ ILC2, and (**D**) GATA3^+^ ILC2 in the heart (*n* = 5 per group). (**E**) Anti-CD45 antibody were i.v. administered into saline- or IL-33-injected mice 3 minutes before hearts were harvested. Intravascular (CD45 i.v.^+^; blue arrows) and interstitial (CD45 i.v. ^-^; red arrows) ILC (Lin^-^Thy1.2^+^) and ILC2 (Lin^-^Thy1.2^+^ST2^+^) were determined by flow cytometry. (**F**) Proportion of intravascular (IV^+^, blue) and interstitial (IV^-^, red) CD45^+^ leukocytes (left), Lin^-^Thy1.2^+^ ILC (middle), and Lin^-^Thy1.2^+^ST2^+^ ILC2 (right) in mouse hearts. The percentage are indicated inside the bars; the numbers below the bars represent the total number of each population in the heart (x 10^3^). Each panel shows the representative data from two independent experiments (*n* = 5 per group). **(G)** Representative plots and gating strategy for ILC2 population identification in hearts from saline or IL-33-treated *ST2^‒/‒^*, *IL33^‒/‒^*, and *IL33^-/+^ST2^-/+^* heterozygote littermates. Dashed lines indicate the isotype control, and solid red lines indicate the ST2 signal intensity. Data are representative of three independent experiments.** (H)** Total cell number of leukocytes, ILC, and ILC2s in the cardiac tissues (n = 3 in each group). **(I)** The heart tissues were harvested and the mRNA were isolated for qRT-PCR analysis of gene expression (n = 3 in each group). *P < 0.05 by one-way ANOVA followed by the Bonferroni multiple comparison post-hoc test. All values are means ± SD. Each dot indicates a biological replicate.

To examine whether ILC2s are localized in mouse cardiac tissue, we performed flow cytometry analysis of isolated suspension cells from mouse hearts. This identified a resident cardiac ILC population expressing surface markers (CD45^+^Lin^-^Thy1.2^+^), which is consistent with the characteristics of ILC2s found in peripheral blood and lung tissues **(Figure 1A and Figure S1A-B)** 31. In normal heart tissues, ST2 and KLRG1 expression in the cardiac resident ILC2s (CD45^+^Lin^-^Thy1.2^+^) was relatively lower than that in lung ILC2s. However, IL-33 treatment increased the marker expression and ILC2 number, indicating cardiac ILC2 population expansion and activation by IL-33 treatment **(Figure 1A, C and Figure S1A-B)**. Moreover, IL-33 treatment increased transcription factor GATA3 expression in the total cardiac ILCs (CD45^+^Lin^-^Thy1.2^+^), suggesting the increased ILC2-committed population upon IL-33 treatment** (Figure 1B, D)**.

In addition, we labeled blood leukocytes using fluorescence-labeled anti-CD45 antibody before hearts were harvested to ensure that cardiac ILC2 is located in the interstitial region. However, CD45 i.v. failed to label cardiac ILC2 at a steady state and minimally labeled IL-33-treated heart ILC2 compared with leukocytes, suggesting that cardiac ILC2 is a tissue-resident leukocyte **(Figure 1E-F)**.

### IL-33-mediated expansion of cardiac ILC2s requires ST2 signaling

We next examined whether IL-33 expands ILC2s via its receptor ST2. The hearts from Saline or IL-33-treated *ST2^‒/‒^*, *IL33^‒/‒^*, and *IL33^-/+^ST2^-/+^* heterozygote littermates were isolated for flow cytometry analysis of ILC2 populations **(Figure 1G)**. IL-33 treatment increased the frequency of cardiac ILC2 (Lin^-^Thy1.2^+^ST2^+^) in heterozygote littermates and *IL33^‒/‒^* mice, but not in *ST2^‒/‒^* mice **(Figure 1G-H)**. ILC2-derived cytokine (*IL5*, *IL13*, and *Areg*) gene expression was also increased by exogenous IL-33 in heterozygote littermates and *IL33^‒/‒^* but not *ST2^‒/‒^* mice **(Figure 1I)**, indicating that IL-33-mediated ILC2 expansion is dependent on ST2 signaling and that *ST2^-/+^* heterozygosity is sufficient for IL-33-mediated ILC2 expansion *in vivo*.

### Endogenous ILC deficiency in *Rag2^‒/‒^IL2Rγc^‒/‒^* mice exacerbates cardiac fibrosis following myocardial injury

**Figure 2:**
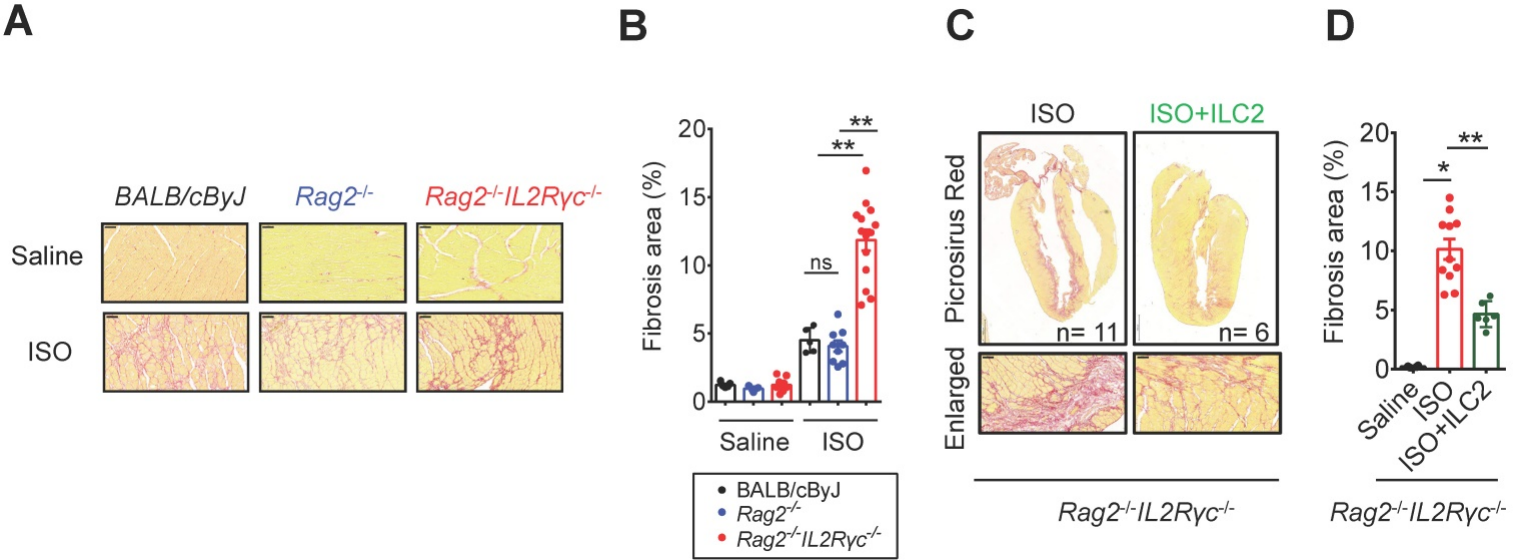
** Deficiency of endogenous ILCs develops exacerbated cardiac fibrosis following injury. (A)** BALB/cByJ, *Rag2*^-/-^, and *Rag2*^-/-^*IL2Rγc*^ -/-^ mice were subcutaneously administered saline or isoproterenol (ISO, 30 mg/kg per day for 3 days). The cardiac tissues were collected for histological analysis on day 4 after the last injection. Representative images of Picrosirius red staining for fibrotic area are shown. Scale bar = 50 µm. **(B)** Quantification of fibrosis area to assess susceptibility to isoproterenol (ISO)-induced cardiac fibrosis. Data are pooled from two independent experiments and expressed as the mean ± SD (*n* = 5-9 per group).** (C)** Picrosirius red staining for fibrotic area. **(D)** Quantification of fibrosis area. *P < 0.05, **P < 0.01 by one-way ANOVA followed by the Bonferroni multiple comparison post-hoc test. All values are means ± SD. Each dot indicates a biological replicate.

As tissue damage, inflammation, and repair responses occur following cardiac injury, the ILC2 population might be altered in the injured heart and contribute to inflammation and tissue reparative responses. To test this hypothesis, we implemented the isoproterenol (ISO)-induced cardiac injury model for catecholamine-induced stress cardiomyopathy 32 in wild type BALB/cByJ mice, *Rag2*^‒/‒^ mice (which lack T and B lymphocytes with preserved ILCs) and *Rag2^‒/‒^IL2Rγc^‒/‒^* mice (which lack T and B lymphocytes and all ILC subgroups) and compared the tissue fibrosis levels to delineate the contribution of ILC2s in the tissue responses following cardiac injury. Histopathological assessment by Picrosirius red staining revealed that ILC-deficient *Rag2^‒/‒^IL2Rγc^‒/‒^* mice were more susceptible to ISO-induced cardiac fibrosis when compared with wild-type BALB/cByJ and ILC-sufficient *Rag2*^‒/‒^ mice **(Figure 2A)**. We did not observe any difference in the fibrosis area between wild-type and *Rag2*^‒/‒^ mice. The average percentage of cardiac fibrosis area was greater in *Rag2^‒/‒^IL2Rγc^‒/‒^* mice than in *Rag2*^‒/‒^ and BALB/cByJ mice **(Figure 2B)**.

To specifically address the role of ILC2s in ISO-induced cardiac fibrosis, we further examined whether adoptive transfer of FACS-sorted ILC2 into *Rag2^‒/‒^IL2Rγc^‒/‒^* mice. Strikingly, reconstitution of FACS-sorted ILC2s via tail vein injection ameliorated ISO-induced cardiac fibrosis in* Rag2^‒/‒^IL2Rγc^‒/‒^* mice, confirming the anti-fibrotic role of ILC2s **(Figure 2C-D)**.

### IL-33 increases cardiac ILC2 accumulation and alleviates ISO-induced cardiac fibrosis

We next determined whether IL-33-mediated ILC2 expansion protects against ISO-induced cardiac fibrosis. We employed BALB/cByJ mice for the ISO-induced fibrosis model as these are more susceptible to ISO-induced cardiac fibrosis 33-35. Immunofluorescence staining to detect endogenous IL-33 expression in the injured cardiac tissues revealed increased endogenous IL-33 expression that was predominantly localized in vimentin+ fibroblasts and in a minor population of CD31^+^ endothelial cells **(Figure S2)**. IL-33^+^ cell number increased and peaked on day 1, being sustained until day 12 following ISO-induced cardiac injury **(Figure S2A-B)**.

To further examine the effect of IL-33 in ISO-induced cardiac fibrosis, the wild-type BALB/cByJ mice were treated with Saline, ISO, or ISO+IL-33 **(Figure S3A)**. The hearts were harvested on day 4 after the last ISO injection for flow cytometry analysis for ILC2 populations and pathological assessments. Notably, flow cytometry analysis revealed no difference in ILC2 frequency in the heart following ISO treatment compared with that for Saline control, suggesting that the increased endogenous IL-33 expression is likely not sufficient to trigger ILC2 expansion **(Figure S3B-C)**. Conversely, exogenous IL-33 treatment increased ILC2 frequency and number in the heart compared to that in the ISO+IL-33 group **(Figure S3B-C)**. Picrosirius red staining for fibrosis area showed that IL-33 co-treatment significantly reduced cardiac fibrosis lesion areas at day 4 following ISO challenge **(Figure S3D)**. This result indicated that IL-33 treatment increased cardiac ILC2 accumulation in the cardiac tissue following ISO-induced fibrosis. In addition, we also showed that prophylactic pre-treatment of IL-33 protects against ISO-induced fibrosis **(Figure S3E-F)**.

**Figure 3:**
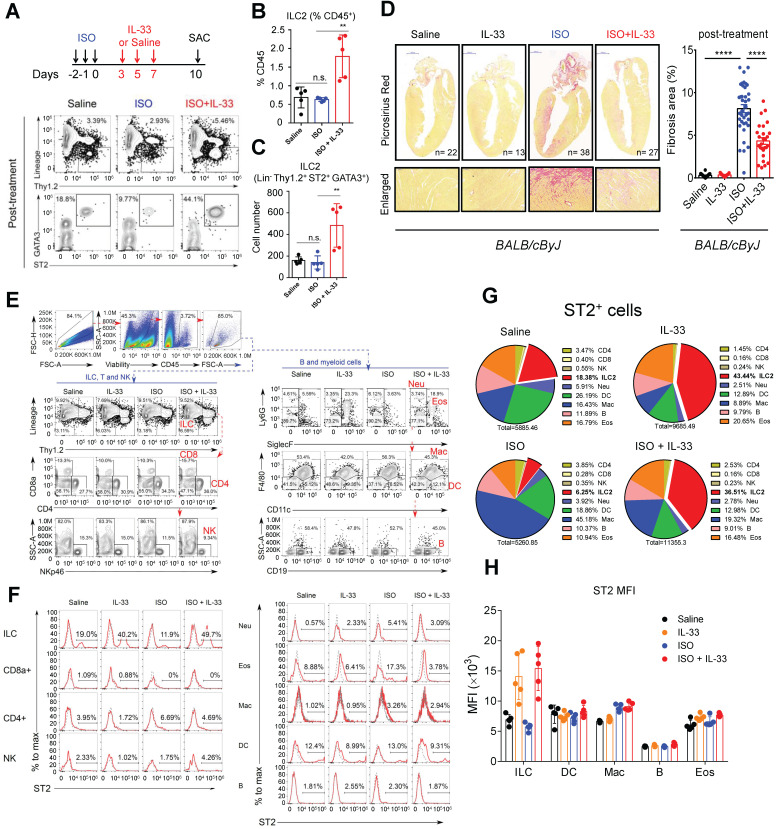
** IL-33 expands cardiac ILC2 cells and reduces ISO-induced cardiac fibrosis. (A)** BALB/cByJ mice were subcutaneously administered saline or ISO followed by saline or IL-33 on days 3, 5, and 7. The heart tissues were harvested on day 10 after the last ISO injection. Flow cytometry analysis for the CD45^+^Lin^-^Thy1.2^+^ ILC2 cells in the cardiac tissues on day 10 after the last ISO injection. **(B)** Frequency of ILC2 among CD45^+^ cells.** (C)** Number of ILC2s per heart. **(D)** Picrosirius red staining and quantification of cardiac fibrosis area. Data are pooled from two independent experiments and expressed as the means ± SD (n = 5-10 per group). Scale bar = 100 µm. *P < 0.05, **P <0.01, ***P <0.001, ****P <0.0001; ns, not significant by one-way ANOVA followed by the Bonferroni multiple comparison post-hoc test. All values are means ± SD. Each dot indicates a biological replicate. **(E)** Gating strategy for each leukocyte population and the comparison of each condition. **(F)** ST2^+^ cell gating for the gated leukocyte population. **(G)** Proportion of ST2-positive leukocytes. **(H)** Mean fluorescence intensity (MFI) of ST2 on the surface of ILC (Lineage^-^ Thy1.2^+^), DC (CD11c^+^), macrophages (F4/80^+^), B cells (CD19^+^), and eosinophils (CD11b^+^ SiglecF^+^).

Next, we examined whether IL-33 post-treatment could reduce ISO-induced cardiac injury to determine the therapeutic potential of IL-33 treatment for established myocardium fibrosis. Mice were administered three doses of ISO challenges followed by three doses of IL-33 or saline on days 3, 5, and 7 post-ISO **(Figure 3A)**. The frequency and cell number of ILC2 in the heart tissue were increased by IL-33 at day 10 following ISO challenge in the post-treatment experiment **(Figure 3B-C)**. Histopathological assessment also showed that IL-33 treatment reduced ISO-induced cardiac fibrosis **(Figure 3D)**. Together, these results suggested an anti-fibrotic effect of IL-33 in ISO-induced cardiac fibrosis.

Furthermore, we analyzed the expression level of ST2 on the cell surface of major leukocytes in the heart using flow cytometry **(Figure 3E-F)**. ILC2 occupied only a small proportion but showed the highest expansion among ST2^+^ leukocytes after IL-33 was administrated **(Figure 3F-G)**. ILC2 also showed the highest ST2 expression level on the cell surface **(Figure 3F-H)**. These indicate that ILC2 is a major recipient in the heart after IL-33 treatment. Additionally, the dominance of ILC2 in ST2^+^ leukocytes can be ascertained in the post-treatment protocol with or without ISO stimuli **(Figure 3E-H)**.

### IL-33 treatment prevents ISO-induced progressive cardiac function impairment

**Figure 4:**
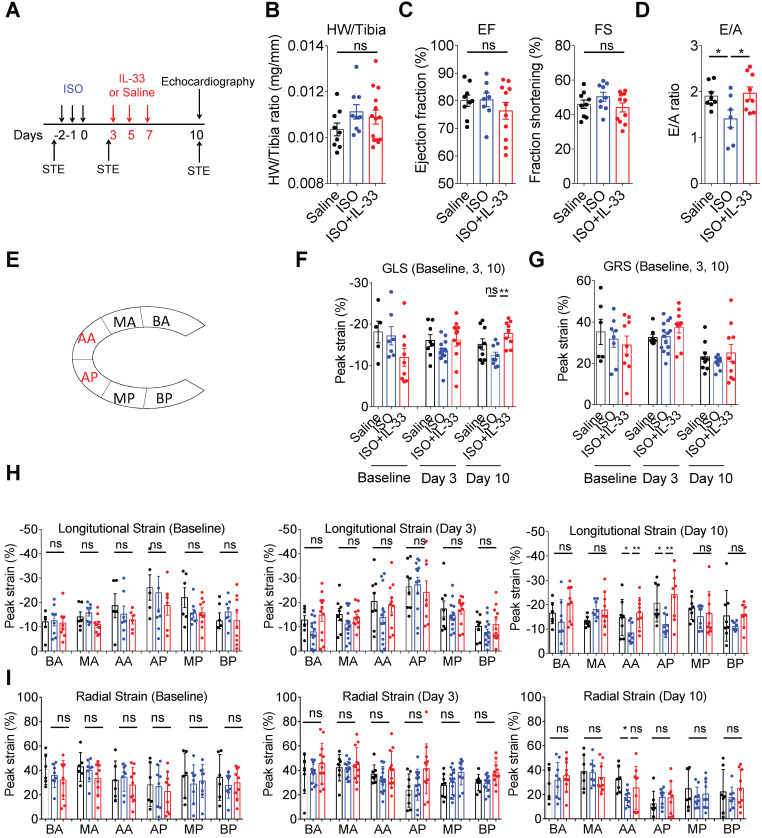
**IL-33 treatment prevents ISO-induced progressive cardiac function impairment. (A)** BALB/cByJ mice were subcutaneously administered isoproterenol (ISO) (60 mg/kg) for three days followed by intraperitoneal Saline or IL-33 (0.5 μg/mouse) treatment on days 3, 5, and 7. Cardiac functions were evaluated by M-mode echocardiography (on day 10), pulse wave Doppler (on day 10), and speckle-tracking echocardiography (STE, on day -3 before ISO challenge as baseline, day 3 after the last ISO injection before IL-33 or saline administration, and day 10 post-ISO challenge) (*n* = 6-10 per group) before the mice were euthanized (SAC). **(B)** Heart weight to tibia length ratio of the mice between groups. **(C)** Analysis of the ejection fraction (EF) and fraction shortening (FS) by M-mode echocardiography. **(D)** Pulse wave Doppler analysis of the ratio of peak velocity of early to late filling of mitral inflow (E/A)** (E)** Schematic overview of anatomical segments in the parasternal long-axis view. Anterior (AA) and posterior (AP) apex; anterior (BA) and posterior (BP) base; anterior (MA) and posterior (MP) mid. **(F)** Quantification of the global longitudinal strain (GLS) and **(G)** global radial strain (GRS) of the mice. **(H)** Quantification of the segmental longitudinal peak strain upon ISO and IL-33 treatment. **(I)** Quantitative analysis of segmental radial peak strain. *P < 0.05 by one-way ANOVA followed by the Bonferroni multiple comparison post-hoc test. All values are means ± SD. Each dot indicates a biological replicate. *P < 0.05, **P<0.01 by one-way ANOVA followed by the Bonferroni multiple comparison post-hoc test.

Traditional M-mode echocardiography and the speckle-tracking echocardiography, which allows more precise analysis of cardiac tissue segmental impairments 36, were performed to evaluate the cardiac contractility changes on day 10 post-ISO challenge **(Figure 4A)**. Analysis of the heart weight to tibial ratio did not show significant cardiac hypertrophy in this model** (Figure 4B)**. Cardiac function analysis by M-mode echocardiography analysis did not show functional impairments on ejection fraction and fraction shortening between groups **(Figure 4C)**. Pulse wave Doppler analysis of the ratio of peak velocity of early to late filling of mitral inflow (E/A) showed a decreased E/A ratio in the ISO group compared with the saline control, whereas this was reversed by IL-33 treatment **(Figure 4D)**. Using speckle-tracking echocardiography, the segmental strains in the groups at days 0 (baseline), 3 (post-ISO and prior to IL-33 or saline injection), and 10 (endpoint) were assessed. Intriguingly, we did not observe any considerable significant difference in the segmental strain or strain rate at baseline and day 3 post-ISO **(Figure 4F-I)**, suggesting that segmental strain is not altered on day 3 despite myocardial injury. Quantification results of the segmental cardiac contractility revealed that ISO treatment markedly decreases the global longitudinal strain, whereas this was reversed by IL-33 treatment on day 10 **(Figure 4F, H)**. The global radial peak strain remained unchanged **(Figure 4G, I).** Further quantification of the segmental longitudinal strain revealed that ISO-induced functional impairment is restricted at the apical segments, including posterior apices (AP) and anterior apices (AA), which was consistent with the pathological findings of endocardium fibrosis on day 10 **(Figure 3D)**. Conversely, IL-33 treatment attenuated the ISO-induced apical segment functional impairment.

### IL-33 treatment reverses the gene transcription profiles in ISO-induced cardiac fibrosis

**Figure 5:**
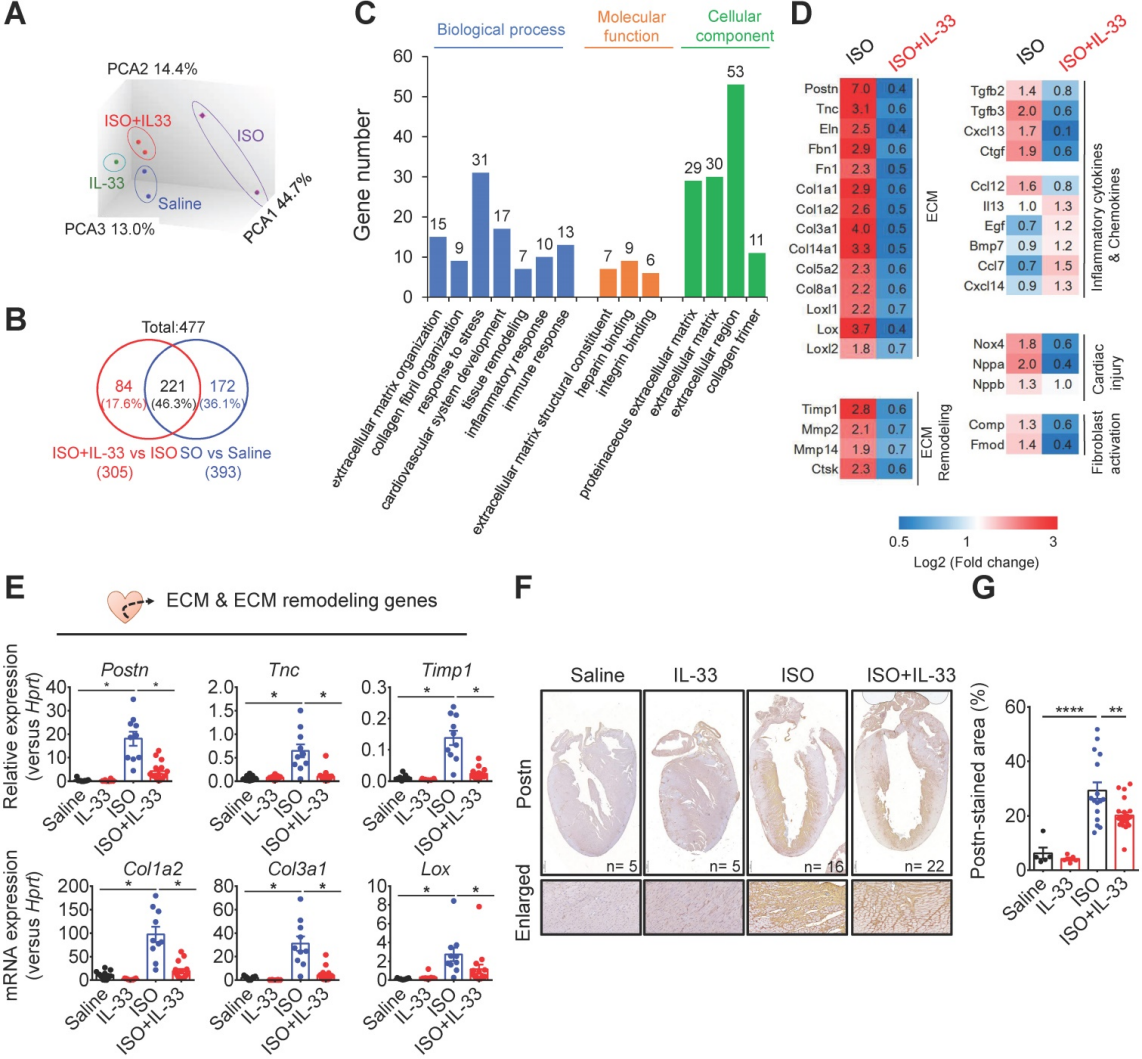
** IL-33 treatment reverses isoproterenol (ISO)-induced differentially regulated gene expression as shown by transcriptome profiling.** BALB/cByJ mice were subcutaneously administered saline or ISO (60 mg/kg per day for 3 days) followed by saline or IL-33 (0.5 μg/mouse, intraperitoneal injection) on day 3, 5, 7. The heart tissues were harvested on day 10 after the last injection for mRNA isolation and microarray analysis. The mRNAs from three hearts were pooled as one sample for transcriptome analysis by Affymatrix microarray. **(A)** Principle component analysis (PCA) for the gene expression profile between groups. **(B)** Number of differentially expressed genes (DEGs) between (ISO vs Saline) and (ISO vs ISO+IL-33) groups. **(C)** Functional annotation clusters identified by gene ontology of DEGs between (ISO vs Saline) and (ISO vs ISO+IL-33) groups. **(D)** Representative cluster DEGs for extracellular matrix (ECM), ECM remodeling enzymes, inflammatory cytokines and chemokines, and cardiac injury-related genes. Fold increase in the ISO and ISO+IL-33 group represents fold change in gene expression of ISO vs Saline and ISO+IL-33 vs ISO group, respectively. **(E)** qRT-PCR validation of the ECM-associated gene expression (*Postn*, *Tnc*, *Timp1*, *Col1a2*, *Col3a1*, and *Lox*) in the cardiac tissues. *P < 0.05 by one-way ANOVA followed by the Bonferroni multiple comparison post-hoc test. (n = 8-10 per group). **(F-G)** Immunohistochemical staining for periostin (Postn) expression and quantification of positive-stained areas in the heart tissue sections. Scale bar = 100 µm. **P < 0.01, ****P < 0.0001 by one-way ANOVA followed by the Bonferroni multiple comparison post-hoc test. All values are means ± SD. Each dot indicates a biological replicate.

To further investigate the mechanisms underlying IL-33-mediated anti-fibrotic responses *in vivo*, we analyzed the changes of the transcriptome profiles in the hearts from Saline-, IL-33-, ISO-, and ISO+IL-33-treated groups by microarray analysis **(Figure 5)**. Principal component analysis revealed that the ISO-induced transcriptome profile alteration was reversed following IL-33 treatment **(Figure 5A)**. Among the 477 differentially expressed genes (DEGs, *P* < 0.05, fold change >1.5 versus Saline), the up- or down-regulation of 221 DEGs (46.3%) by ISO treatment was reversed by IL-33 **(Figure 5B)**. Functional annotation of the IL-33-reversed DEGs revealed clusters enriched for ECM organization, collagen fibril organization, and immune responses **(Figure 5C)**. The expression of IL-33-regulated DEGs associated with ECM, ECM remodeling, inflammatory cytokines and chemokines, and cardiac injury markers was altered upon ISO challenge albeit reversed by IL-33 treatment **(Figure 5D)**.

To validate the microarray data, we performed qRT-PCR to analyze ECM and ECM remodeling-associated gene expressions (**Figure 5E**). Consistent with the transcriptome analysis results, IL-33 treatment reduced ISO-induced gene expression associated with fibroblast activation (*Postn* and *Tnc*) 37, 38, ECM production (*Col1a2* and *Col3a1*) 39, collagen crosslinking (*LOX*) 40, and ECM remodeling (*Timp1*). This suggested that exogenous IL-33 treatment attenuated stress-induced cardiac fibrosis and reversed the transcriptional profile related to cardiac remodeling and fibrosis (**Figure 5E**). In addition to the reduced collagen deposition **(Figure 3D)**, immunohistochemical staining further confirmed that IL-33 treatment reduced ISO-upregulated cardiac periostin protein levels, which is associated with cardiac fibroblast activation 37 **(Figure 5F-G)**. These results together demonstrated that IL-33 post-treatment alleviated adverse cardiac fibrotic responses by reducing ECM and ECM remodeling-associated gene expressions.

### IL-33 treatment reduces cell death following ISO-induced cardiac fibrosis

We next confirmed whether IL-33 attenuated ISO-induced cell death in cardiac tissues by using terminal deoxynucleotidyl transferase dUTP nick end labeling (TUNEL) immunofluorescence staining, a marker of cell death/or apoptosis. ISO-induced cardiac injury increased the number of TUNEL^+^ cells in the cardiac tissues, whereas this was reduced by IL-33 treatment **(Figure S4A-B)**. Cell DNA damage marker γH2AX expression was increased in the ISO groups, whereas IL-33 reduced the γH2AX^+^ cell number **(Figure S4D-E)**. Additionally, to distinguish between cardiomyocytes and non-cardiomyocytes, we determined the percentages of cTNI^+^TUNEL^+^ cardiomyocytes and cTNI^-^TUNEL^+^ non-cardiomyocytes. The same approach was used to distinguish between cTNI^+^γH2AX^+^ cardiomyocytes and cTNI^-^γH2AX^+^ non-cardiomyocytes. Following ISO challenge, we found that TUNEL^+^ cardiomyocytes were approximately 25% of the total number of TUNEL^+^ cells **(Figure S4C)** and that γH2AX^+^ cardiomyocytes were approximately 60% of the total number of γH2AX^+^ cells **(Figure S4F)**. Although IL-33 did not specifically reduce the percentage of TUNEL^+^ or γH2AX^+^ cardiomyocytes, IL-33 treatment reduced the total number of TUNEL^+^ or γH2AX^+^ cells. This suggests that IL-33 reduced the cell death responses following myocardial injury.

### IL-33 increases type 2 cytokines (IL-5 and IL-13), BMP-7, IL-10, and G-CSF in ILC2s

**Figure 6:**
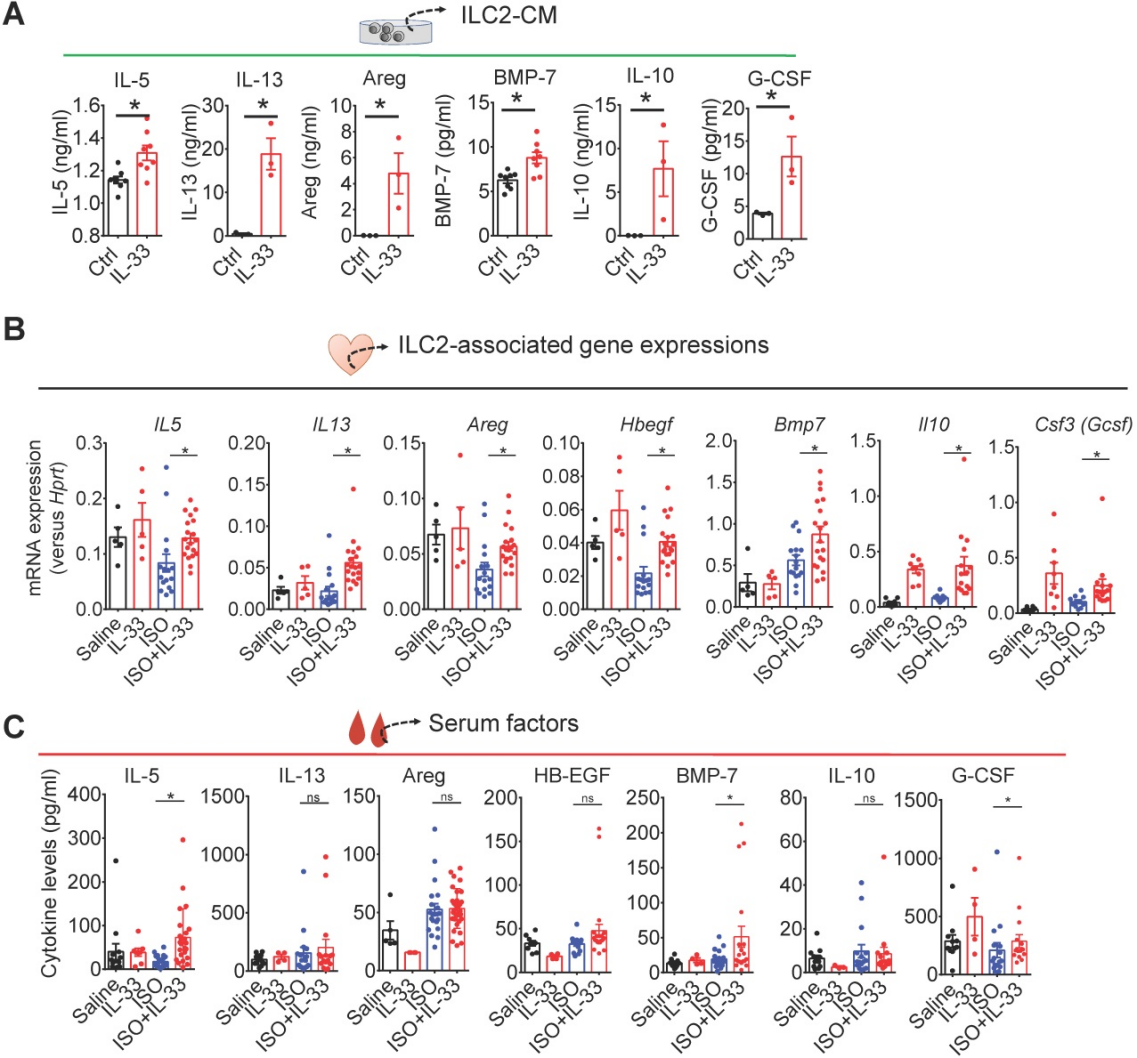
** IL-33 treatment upregulates ILC2-associated factors. (A)** ELISA analysis of cytokine levels in conditioned medium from ILC2 cells cultured in Ctrl (IL-2+IL-7) and IL-33 (IL-2+IL-7+IL-33) media. Each dot indicates a biological replicate. *P < 0.05 by nonparametric Mann-Whitney test. **(B)** qRT-PCR analysis of ILC2-associated gene expression in cardiac tissues. *P < 0.05 by one-way ANOVA followed by the Bonferroni multiple comparison post-hoc test. Each dot indicates a biological replicate. Data are pooled from two independent experiments and expressed as the mean ± SD (*n* = 5-10 per group). **(C)** Serum cytokine levels of mice treated with Saline, IL-33, ISO, or ISO+IL-33. *P < 0.05 by one-way ANOVA followed by the Bonferroni multiple comparison post-hoc test. Data pooled from three independent experiments and expressed as the mean ± SD (*n* ≥ 3 per group). Each dot indicates a biological replicate.

We next tested the hypothesis that ILC2-derived factors contribute to IL-33-mediated effector functions in the injured myocardium. We collected and analyzed cytokine levels in the supernatants from FACS-sorted ILC2s cultured for 48 h with control (IL-2+IL-7) or activation medium (IL-2+IL-7+IL-33). IL-5, IL-13, and Areg levels were higher in the supernatants following IL-33 stimulation **(Figure 6A)**. Additionally, we also found that IL-33 increased the BMP-7, IL-10, and G-CSF levels in ILC2s **(Figure 6A)**.

### IL-13 is required for IL-33-mediated anti-fibrotic response by enhancing alternatively activated M2 macrophages (M2ϕ)

**Figure 7:**
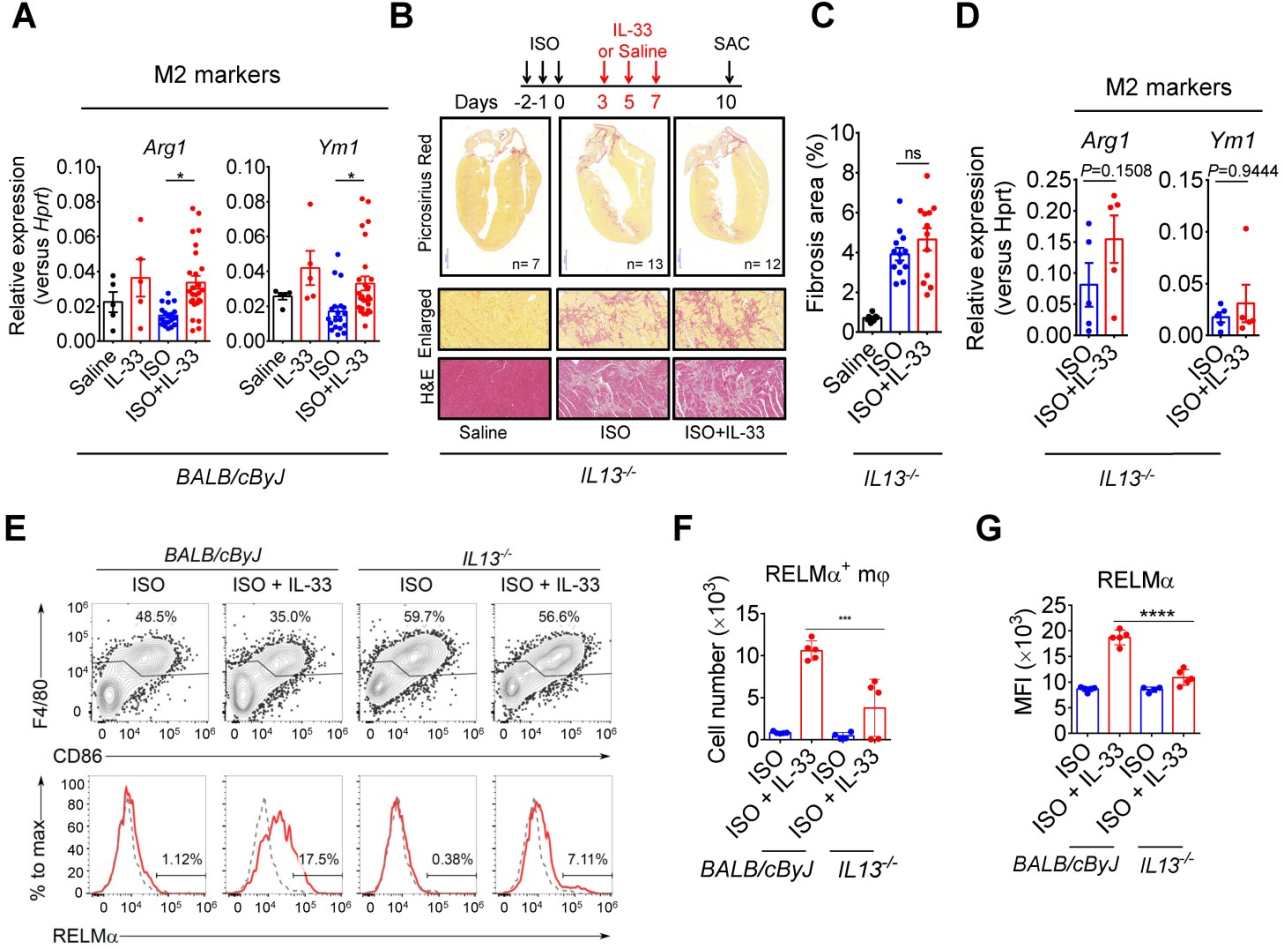
** IL-13 is required for IL-33-mediated anti-fibrotic responses through the activation of alternatively activated macrophages (M2ϕ). (A)** qRT-PCR validation of M2ϕ gene expression (*Arg1* and *YM1*) in cardiac tissues. Data are pooled from two independent experiments and expressed as the means ± SD (n = 5-10 per group). *P < 0.05 by one-way ANOVA followed by the Bonferroni multiple comparison post-hoc test. **(B)** *IL13*^-/-^ mice were subcutaneously administered with isoproterenol (ISO) (60 mg/kg) for three days then intraperitoneally administered with Saline or IL-33 (0.5 μg/mouse) on days 3, 5, and 7. The cardiac tissues were collected for Picrosirius red staining of the fibrotic area. Scale bar = 100 µm. **(C)** Quantification of the fibrosis area in the heart sections. ns, not significant. Data are expressed as the means ± SD. ns, not significant. **(D)** qRT-PCR analyses of M2ϕ gene expression (*Arg1* and *Ym1*) in the cardiac tissues (n=3 in each group). **(E)** Intracellular flow cytometry analysis for CD45^+^F4/80^+^ macrophages and M2ϕ marker RELMα in the heart of wild-type and *IL13^-/-^* mice. **(F)** Number of RELMα^+^ macrophages. **(G)** Mean-fluorescence intensity (MFI) of RELMα in macrophages (*n* =5 per group). ***P<0.001, ****P<0.0001 by one-way ANOVA followed by the Bonferroni multiple comparison post-hoc test. All values are means ± SD. Each dot indicates a biological replicate.

As IL-13 is a crucial downstream effector of ILC2, we evaluated whether IL-13 is required for IL-33-mediated anti-fibrotic responses. In wild-type BALB/cByJ mice, IL-33 treatment increased M2ϕ-associated markers (*Arg1* and *Ym1*) in ISO+IL-33 hearts compared with those in the ISO group **(Figure 7A)**. However, *IL13*-deficiency abolished IL-33-mediated M2 marker activation and IL-33-mediated anti-fibrotic effects** (Figure 7B-D)**. In addition, we directly assayed RELMα, an M2ϕ marker 42, in the macrophage population using flow cytometry (**Figure 7E**) and validated that cardiac M2ϕ conversion was abolished in* IL13*-deficient mice after ISO+IL-33 treatment **(Figure 7F-G)**. These results indicated that IL-13 is a crucial downstream mediator for M2ϕ polarization for optimal anti-fibrotic response following IL-33 treatment.

### EGFR signaling is required for optimal IL-33 cardiac protective function

*In vitro*, we found that IL-33-activated ILC2s produce EGF ligands Areg **(Figure 6A)**. To further confirm the contribution of EGFR signaling in IL-33-mediated function, we investigated whether blocking EGFR signaling by the EGFR inhibitors geftinib or afatinib 43 abolished IL-33-mediated anti-fibrotic function. Both inhibitors attenuated IL-33-mediated pro-survival effects as qRT-PCR analysis showed that IL-33 treatment increased the BCL2/BAX ratio, indicated increased cell survival and reduced apoptosis in the cardiac tissues **(Figure S5A)**. EGFR signaling inhibition, however, abolished IL-33-mediated pro-survival signaling **(Figure S5B)** and anti-fibrotic function **(Figure S5C)**.

### BMP-7 is a novel ILC2-derived anti-fibrotic factor that inhibits TGF-β1-induced CF activation

We next examined whether ILCs are required for IL-33-induced IL-5 or BMP-7 *in vivo*. Strikingly, IL-33 treatment increased IL-5 levels in wild-type BALB/cByJ **(Figure S6C)** and Rag2*^‒/‒^* mice **(Figure S6)** but not in ILC-deficient *Rag2^‒/‒^IL2Rγc^‒/‒^* mice **(Figure S6)**, indicating that ILCs contribute to IL-33-induced circulating IL-5 *in vivo*. However, the induction of circulating BMP-7 by IL-33 was abolished in *Rag2^‒/‒^* mice and *Rag2^‒/‒^IL2Rγc^‒/‒^* mice, indicating that cells other than ILC2 also contribute to the elevation of circulating BMP-7* in vivo*. Nevertheless, ILC2-derived BMP-7 protein level is elevated by IL-33 *in vitro* **(Figure 6A),** and BMP-7 might function as a local cytokine within the myocardium.

**Figure 8:**
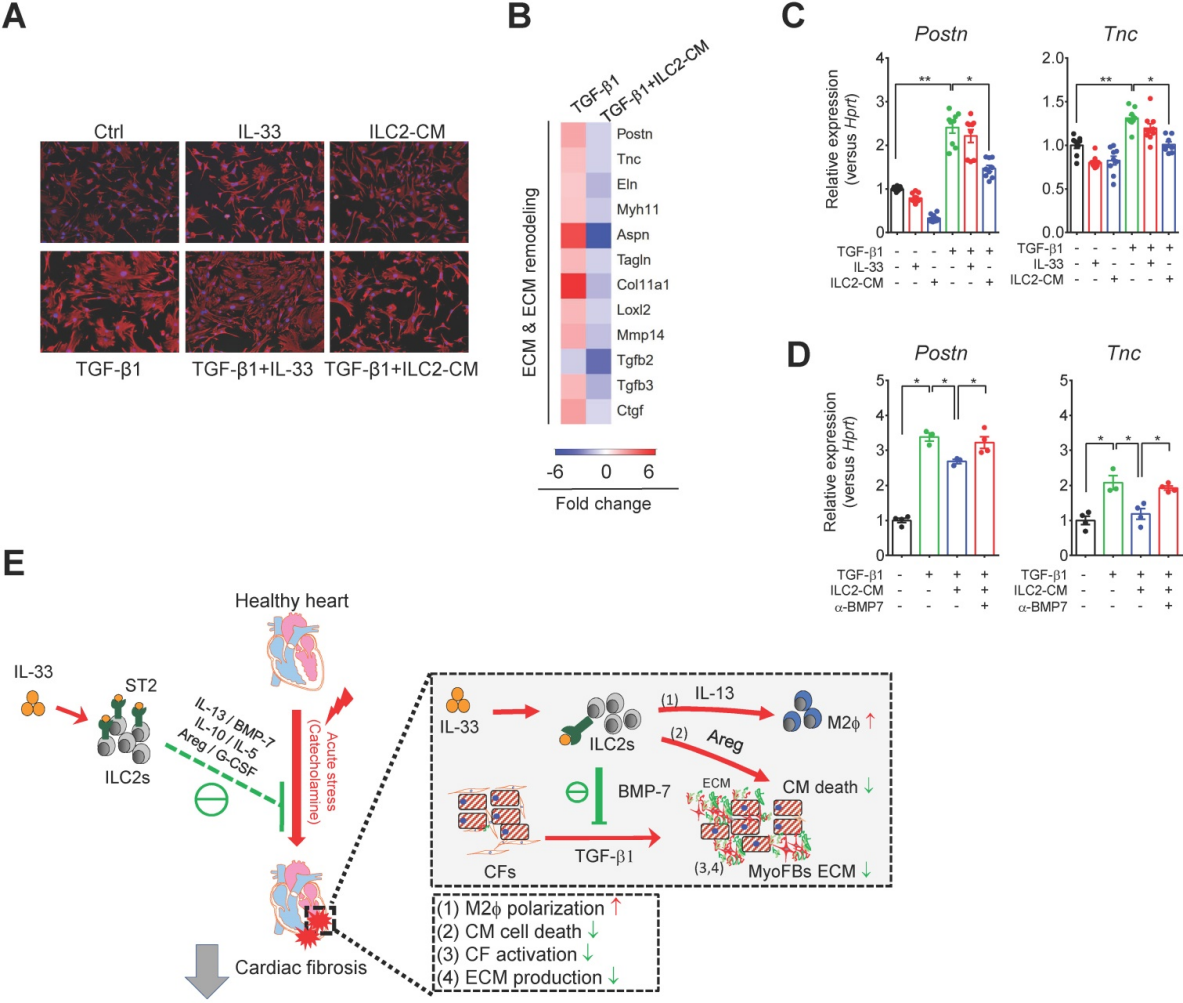
** ILC2-derived factors inhibit TGF-β1-induced cardiac fibroblast activation. (A)** Primary mouse cardiac fibroblasts were cultured with Saline IL-33 (30 ng/mL) or ILC2-conditioned media (ILC2-CM, 1:100 dilution) for 24 h in the presence or absence of TGF-β1. Cell morphology of the mouse cardiac fibroblasts was analyzed by immunostaining for vimentin (red). Cell nuclei were stained by DAPI (blue). **(B)** Transcriptome analysis for the gene expressions associated with ECM and ECM remodeling. Fold increase in the TGF-β1 and TGF-β1+ILC2-CM group represents fold change in gene expression of TGF-β1 vs Saline and TGF-β1+ILC2-CM vs TGF-β1 group, respectively. **(C)** mRNA expression of *Postn* and *Tnc* was analyzed by qRT-PCR.** (D)** qRT-PCR analysis on TGF-β1-induced *Postn* and *Tnc* in the presence or absence of BMP-7 neutralization antibody (1 μg/mL). *P < 0.05, **P < 0.01, ns, not significant by one-way ANOVA followed by the Bonferroni multiple comparison post-hoc test. All values are means ± SD. Each dot indicates a biological replicate. **(E)** Working model for IL-33-mediated ILC2s expansion for protection against cardiac fibrosis. IL-33 treatment expands and activates cardiac ILC2s to secrete soluble factors including IL-13, IL-5, Areg, IL-10, G-CSF, and BMP-7. IL-13 is crucial for IL-33-mediated cardiac protective function by regulating M2 macrophage (M2ϕ) polarization. ILC2-derived BMP-7 alleviates TGF-β1-induced cardiac fibroblast activation. Taken together, in combination with the increased M2 polarization, reduced ECM production, reduced CF activation, and reduced cell death within the myocardium, IL-33 alleviates cardiac fibrosis via the expansion of ILC2 and the effector functions of ILC2-derived paracrine factors.

To further test the hypothesis that ILC2-derived BMP-7, an anti-fibrosis factor secreted by ILC2s upon IL-33 stimulation 44, 45, could antagonize TGF-β-induced CF activation, we isolated primary mouse CFs and cultured the cells with Saline, IL-33, or ILC2-CM for 24 h in the presence or absence of TGF-β1. Cell morphology analysis by vimentin staining revealed that CF co-culture with ILC2-CM attenuated TGF-β1-induced morphological changes toward myofibroblast phenotypes **(Figure 8A)**. Microarray analysis revealed that TGF-β1-induced the gene expressions associated with ECM and ECM remodeling, whereas co-culture of mouse CF with ILC2-CM reduced TGF-β1-induced gene expressions associated with ECM and ECM remodeling **(Figure 8B)**.

qRT-PCR analysis showed that TGF-β1, but not IL-33 or ILC2-CM alone, stimulated mouse CF activation as shown by increased fibroblast marker (*Postn* and *Tnc*) transcript levels compared with those of Saline control **(Figure 8C)**. CF co-culture with ILC2-CM, but not with IL-33, reduced TGF-β1-induced *Postn* and *Tnc* gene expression **(Figure 8C)**. Blocking of BMP-7 by neutralizing antibody abolished the inhibitory effect of ILC2-CM on TGF-β1-induced *Postn* and *Tnc* **(Figure 8D)**, suggesting an essential role of BMP-7 on anti-fibrotic function in ILC2-CM. Together, our results demonstrated that IL-33-responsive ILC2-derived factors concurrently contribute to IL-33-mediated anti-fibrotic responses for alleviation of adverse myocardial fibrosis.

## Discussion

In the present study, we demonstrated the evidence of presence of IL-33-responsive ILC2 cells in cardiac tissue. We identified the presence of a resident cardiac ILC2 population that shares similar characteristics of cell surface marker (Lin^-^Thy1.2^+^KLRG1^+^ST2^+^) and transcriptional factor (GATA3^+^) expression with other tissue resident ILC2s 31 . IL-33 treatment induced cardiac ILC2 cell expansion in an ST2-dependent manner. Moreover, ILC-deficient *Rag2^‒/‒^IL2Rγc^‒/‒^* mice were more susceptible to ISO-induced cardiac fibrosis compared with ILC-sufficient *Rag2^‒/‒^* mice, implying that impaired ILC function might attribute to detrimental cardiac remodeling and tissue fibrosis. The protective role of ILC2s was further confirmed by adoptive transfer *Rag2^‒/‒^IL2Rγc^‒/‒^* mice following ISO challenge. Transcriptome analysis revealed that IL-33 attenuated ECM, ECM remodeling, and CF activation-associated gene expression. IL-33-activated ILC2s constitute sources of type 2 cytokines, EGF ligands, and the anti-fibrotic factor BMP-7. We further revealed that IL-33-responsive ILC2s contribute to IL-33-mediated tissue reparative functions involving EGFR signaling, IL-13-mediated M2 macrophage activation, and BMP-7-mediated anti-fibrotic responses. These factors concurrently facilitate IL-33-mediated cardiac protective effects in alleviating cardiac fibrosis.

Notably, although endogenous IL-33 was upregulated in the injured hearts, ILC2 frequency was not altered; indicating that the local IL-33 elevation was not sufficient to trigger ILC2 expansion. Additionally, *IL33*- or *ST2*-deficiency did not affect cardiac-resident ILC2 abundance, suggesting that IL-33/ST2 signaling primarily mediates ILC2 expansion and activation rather than maintaining homeostasis. Recently, IL-33 was shown to promote egress of bone marrow ILC2s 46. As exogenous IL-33 activated systemic responses, it is not restricted to activation of cardiac ILC2s. Moreover, it is not clear whether IL-33-promoted ILC2 activation in other organs contributes to the cardiac protective functions by increasing the systemic serum factors. It is also unclear whether IL-33-activated bone marrow or spleen ILC2s migrate and accumulate in the injured hearts. The precise pathophysiological functions of the cardiac-resident ILC2s in homeostasis and disease conditions thus require further investigation.

ST2 signaling underlies IL-33-mediated cardiac protective function in pressure overload 24. Here, we further confirmed that pharmacological inhibition of EGFR signaling abolished IL-33-mediated pro-survival effects and blunted IL-33-mediated anti-fibrotic function* in vivo*. The EGFR ligand Areg exerts cardioprotective functions in a mouse model of myocardial infarction 47; Treg-derived Areg also promotes myocardial repair following injury 48, 49. Our results suggested that IL-33 likely elicited pro-survival signaling in cooperation with Areg- or HB-EGF-mediated EGFR signaling to promote cardiomyocyte survival following catecholamine-induced cardiac stress injury.

IL-13, a crucial mediator to promote M2ϕ activation, may mediate the effector function of ILC2-associated type 2 immune responses 50, 51. Our results revealed that IL-13 is required for IL-33-mediated M2ϕ activation *in vivo*, which may imply that the IL-33/ILC2/IL-13 axis contributes to tissue repair via M2ϕ activation. Although IL-13 activation exerts both beneficial and detrimental functions in tissue repair and fibrosis, our results demonstrated that IL-13-mediated M2ϕ activation is required for optimal IL-33 cardiac protection. Further study to dissect the optimal time window for IL-33-based therapy for treating tissue fibrosis might be crucial as excessive ILC2 or IL-13 signaling activation in the context of allergic conditions might lead to adverse tissue remodeling and detrimental side effects 52-54. In a recent report showed that ILC2-dependent activation of eosinophils via IL-5 contributes to the development of pericarditis 54. In our model, we also observed increased number of eosinophils in the heart after IL-33 treatment **(Figure 3G)**, whereas we did not observe clinical or histological phenotypes of myocarditis according to echocardiography and histological assessments. This discrepancy might due to the difference in the dose of IL-33 administered. In addition to their pro-inflammatory role, eosinophils have been reported to be beneficial in the repairing process of the injured myocardium 55, 56. Recently, the regulatory eosinophil population has been shown to ameliorate disease onset in a rheumatoid arthritis model 57. These suggest a context dependent and bi-functional effect of ILC2 in balancing excessive inflammatory responses and inflammation resolution. Further study is required to elucidate the regulatory role of the ILC2/eosinophils axis in the pathophysiology of pericarditis, myocardial injury, cardiac fibrosis, and resolution of inflammation. We also identified IL-10 and G-CSF as potential mediators involved in ILC2-mediated immunomodulatory functions. The anti-inflammatory cytokine IL-10 promotes inflammation resolution 58. G-CSF is protective in myocardial infarction 59, 60. Whether IL-33-mediated cardiac protective effects require these factors to exert their effector function in the tissue reparative response requires further investigation.

Previous studies using deep RNA sequencing have identified several markers including BMP-7 uniquely enriched in ILC2 compared to other innate lymphoid cells 61-63. However, the role of BMP-7 in ILC2-mediated effector function in homeostasis and diseased conditions are poorly investigated. In this study, we show that BMP-7 is present in IL-33-activated ILC2s and counteracts TGF-β1-induced CF activation. ILC2-sufficient* Rag2^‒/‒^*and ILC-deficient *Rag2^‒/‒^IL2Rγc^‒/‒^* mice did not respond to IL-33 treatment **(Figure S6)**, suggesting that cells other than ILC2s contributed to the elevation of circulating BMP-7 levels. The local ILC2-derived BMP-7 protein elevated by IL-33 might function as an anti-fibrotic paracrine factor within the myocardium. A recent study showed that circulating ILC2 are not able infiltrate into the heart tissues 22, which may implicate the expansion of tissue resident ILC2s or increase the production of soluble mediators attribute to the major effector functions of local expanded ILC2s. Further study is necessary to dissect the major sources of IL-33-induced BMP-7 and the role of the IL-33/ILC2/BMP7 axis in alleviating adverse cardiac fibrosis.

CF activation and excessive ECM production contribute to the process of tissue fibrosis. In our present study, transcriptome analysis revealed that IL-33 treatment reduced ISO-induced ECM and ECM remodeling-associated gene expression. However, IL-33 alone did not alter TGF-β1-induced *Postn* and *Tnc* gene expressions in CFs *in vitro*, suggesting that IL-33-mediated paracrine factors from ILC2s contribute to the reduced CF activation toward myofibroblast phenotype.

Here, our results demonstrated that ILC2s are present in the cardiac tissue and that IL-33 mediates its cardiac protective function through expansion of ILC2s in the heart. Using a mouse model of cardiac fibrosis, we showed that IL-33-mediated ILC2 expansion is responsible for optimal anti-fibrotic functions via ILC2-derived factors **(Figure 8E)**. Harnessing IL-33-mediated immunomodulatory function thus constitutes a promising strategy to promote tissue repair and alleviate cardiac fibrosis following acute cardiac injury.

## Supplementary Material

Supplementary figures and tables.

## Abbreviations

AA: anterior apices
AP: posterior apices
Areg: amphiregulin
CF: cardiac fibroblast
DEGs: differentially expressed genes
E/A: early to late filling of mitral inflow
ECM: extracellular matrix
EDD: end-diastolic dimension
EGFR: epidermal growth factor receptor
ESD: end-systolic dimension
FACS: fluorescence-activated cell sorter
IL: interleukin
ILC2: group 2 innate lymphoid cell
ISO: isoproterenol
KCGMH: Kaohsiung Chang Gung Memorial Hospital
NLAC: National Laboratory of Animal Center
PBS: phosphate-buffered saline
qRT-PCR: quantitative real-time polymerase chain reaction
sST2: soluble form
ST2L: full-length transmembrane form
TUNEL: terminal deoxynucleotidyl transferase dUTP nick end labeling
