# Supplementary material for: Group 2 innate lymphoid cells contribute to IL-33-mediated alleviation of cardiac fibrosis
Source: Theranostics. 2021 Jan 1;11(6):2594–611. doi: 10.7150/thno.51648 (PMC7806479; doi:10.7150/thno.51648)
Supplement: Supplementary file 1 — Supplementary figures and tables. [file thnov11p2594s1.pdf]

Supplementary information

Table S1. Antibodies used for flow cytometry and tissue/cell staining

Antibody/reagent	Clone	Concentration (µg/ml)	Supplier (Cat#)
**Biotin anti-F4/80**	REA126	FC (3.75)	Mitenyi Biotec 130-116-514
**PerCP/Cy5.5 anti-CD45**	30-F11	FC (1)	Biolegend 103132
**FITC anti-CD3ε**	145-2C11	FC (2.5)	Biolegend 100306
**FITC anti-CD11b**	M1/70	FC (2.5)	Biolegend 101206
**FITC anti-CD11c**	N418	FC (2.5)	Biolegend 117306
**FITC anti-CD19**	6D5	FC (2.5)	Biolegend 115506
**FITC anti-CD49b**	DX5	FC (2.5)	Biolegend 108906
**FITC anti-F4/80**	BM8	FC (2.5)	Biolegend 123108
**FITC anti-FcεRI**	MAR-1	FC (2.5)	Biolegend 134306
**eFlour 450 anti-Thy1.2**	53-2.1	FC (4)	Thermo Fisher 48-0902-82
**PE anti-ST2**	U29-93	FC (5)	BD 566311
**PE anti-RELMα**	DS8RELM	FC (1)	Thermo Fisher 12-5441-80
**PE/Cy7 anti-CD8a**	53-6.7	FC (1)	Biolegend 100722
**PE/Cy7 Streptavidin**	N/A	FC (2)	Thermo Fisher 25-4317-82
**APC anti-KLRG1**	2F1	FC (2)	Biolegend 138411
**APC anti-SiglecF**	S17007L	FC (2)	Biolegend 155507
**APC anti-CD11c**	N418	FC (1)	Biolegend 117310
**APC anti-NKp46**	29A1.4	FC (1)	Biolegend 137607
**Alexa Fluor 647 anti-GATA3**	L50-823	FC (1:5)	BD 560068
**APC/Cy7 anti-CD4**	GK1.5	FC (1)	Biolegend 100413
**APC/Cy7 anti-CD19**	6D5	FC (1)	Biolegend 115529
**APC/Cy7 anti-CD45**	30-F11	FC (1)	BD 557659
**BV421 anti-CD86**	GL-1	FC (2)	Biolegend 105032
**BV421 anti-GATA3**	16E10A23	FC (1:20)	Biolegend 653814
**BV421 anti-SiglecF**	S17007L	FC (2)	Biolegend 155509
**Fixable Viability Dye eFluor 506**	N/A	FC (1:250)	Thermo Fisher 65-0866-14
**Fixable Viability Dye eFluor 780**	N/A	FC (1:250)	Thermo Fisher 65-0865-14
**Anti-Cardiac Troponin I**	4C2	IF (1:200)	Abcam, ab10231
**Alexa Fluor 647 WGA**	N/A	IF (1:200)	W32466,
**Biotin-Isolectin B4**	N/A	IF (1:100)	Invitrogen, I21414
**Anti-IL-33**	N/A	IF (1:200)	R&D Systems, AF3626
**Anti-Phospho-Histone H2AX (Ser139)**	20E3	IF (1:200)	Cell Signaling, 9718
**Anti-Vimentin**	EPR3776	IF (1:200)	Abcam, ab92547
**Anti-Periostin**	N/A	IHC (1:200)	Sino Biological, 50450-RP02
**Anti-BMP7**	4E7	Neu (1.0)	Arigo, ARG56924

Table S2. Primers used for qRT-PCR.

Gene	Species	Sequence (5'-3')
IL5	Mouse	Forward: TCAGGGGCTAGACATACTGAAG<br>Reverse: CCAAGGAACTCTTGCAGGTAAT
IL13	Mouse	Forward: CCTGGCTCTTGCTTGCCTT<br>Reverse: GGTCTTGTGTGATGTTGCTCA
Areg	Mouse	Forward: GCAGATACATCGAGAACCTGG<br>Reverse: CTGCAATCTTGGATAGGTCCTTG
Hbegf	Mouse	Forward: TTTGGAGAGTCCTTTGCAGA<br>Reverse: TGTGACAATGAGATTCCTTGTG
Bmp7	Mouse	Forward: GATTTCAGCCTGGACAACGAG<br>Reverse: GGGCAACCCTAAGATGGACAG
IL10	Mouse	Forward: GCTCTTACTGACTGGCATGAG<br>Reverse: CGCAGCTCTAGGAGCATGTG
Csf3	Mouse	Forward: CCTGGAGCAAGTGAGGAAGA<br>Reverse: CAGCTTGTAGGTGGCACACA
Bcl2	Mouse	Forward: CCTGTGGATGACTGAGTACC<br>Reverse: GAGACAGCCAGGAGAAATCA
Bax	Mouse	Forward: GTTTCATCCAGGATCGAGCAG<br>Reverse: CATCTTCTTCCAGATGGTGA
Arg1	Mouse	Forward: CCAGAAGAATGGAAGAGTCAGTGT<br>Reverse: GCAGATATGCAGGGAGTCACC
Ym1	Mouse	Forward: CAAGTTGAAGGCTCAGTGGCTC<br>Reverse: CAAATCATTGTGTAAAGCTCCTCTC
Postn	Mouse	Forward: GAACGAATCATTACAGGTCC<br>Reverse: GGAGACCTCTTTTTGCAAGA
Tnc	Mouse	Forward: GCTACTGCCAGGCATCTTTC<br>Reverse: GAAGCTCCCACTGGACTCTG
Timp1	Mouse	Forward: GAGAAACCAGCTTGGAACCAG<br>Reverse: GGGGCCATCATGGTATCTGC
Col1a2	Mouse	Forward: ACTCAGCCACCCAGAGTGGAA<br>Reverse: TTGACAGGTTGGGCCTGGA
Col3a1	Mouse	Forward: GCACAGCAGTCCAACGTAGA<br>Reverse: GCACAGCAGTCCAACGTAGA
Lox	Mouse	Forward: TCTTCTGCTGCGTGACAACC<br>Reverse: GAGAAACCAGCTTGGAACCAG

Table S3. Reagents used in this study

Reagent	Supplier (Cat#)
FBS	Gibco (10437-028)
DMEM/F12	Gibco (11330-032)
10X Trypsin-EDTA (0.5%)	Gibco (15400-054)
Collagenase Type II	Worthington (LS004177)
Penicillin/Streptomycin	Gibco (15140122)
L-Ascorbic acid	Sigma (A4544)
Mouse recombinant IL-33	BioLegend (580502)
Human recombinant TGF-β	PeproTech (100-21C)
High-Capacity RNA-to-cDNA™ Kit	Applied Biosystems (4387406)
TRIzol™ Reagent	Invitrogen™ (15596026)
Quick-RNA 96 Kit	ZYMO (R1052)
Quantinova SYBR Green PCR Kit	Qiagen (208054)
Galetin	Sigma (G9136)
TUNEL (In Situ Cell Death Detection Kit, Fluorescein)	Roche (11684795910)
Picro Sirius Red Stain Kit	Abcam (ab150681)
DL-Isoproterenol Hemisulfate	Santa Cruz (sc-294398)
Mouse IL-5 ELSIA	R&D Systems (DY405-05)
Mouse IL-13 ELISA	R&D Systems (DY413-05)
Mouse IL-10 ELISA	R&D Systems (DY417-05)
Mouse HB-EGF ELISA	R&D Systems (DY8239-05)
Mouse Amphiregulin ELISA	R&D Systems (DY-989)
Mouse G-CSF ELISA	R&D Systems (DY414-05)
Mouse BMP-7 ELISA	Invitrogen (# EMBMP7)
MILLIPLEX MAP Mouse Cytokine/Chemokine Magnetic Bead Panel - Immunology Multiplex Assay	Merck (MCYTOMAG-70K)

A

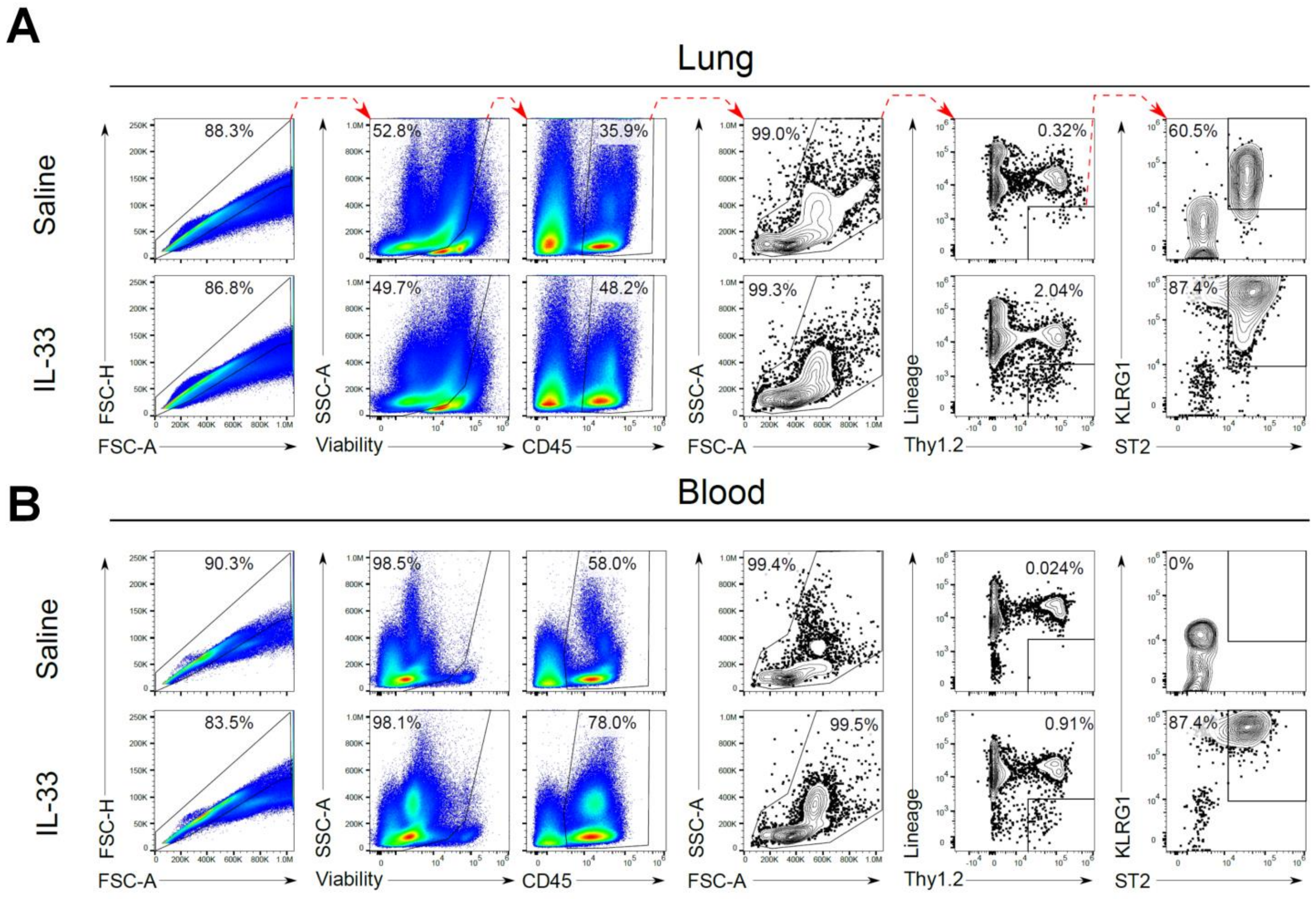

Figure S1. IL-33 increases the cardiac ILC2 population via ST2. Wild-type C57BL/6 mice were treated with saline or IL-33 (2 μg mouse^{-1}day^{-1} for 5 days). **(A-B)** The lung tissues and peripheral blood from the mice were harvested for flow cytometry analysis of the cell surface markers.

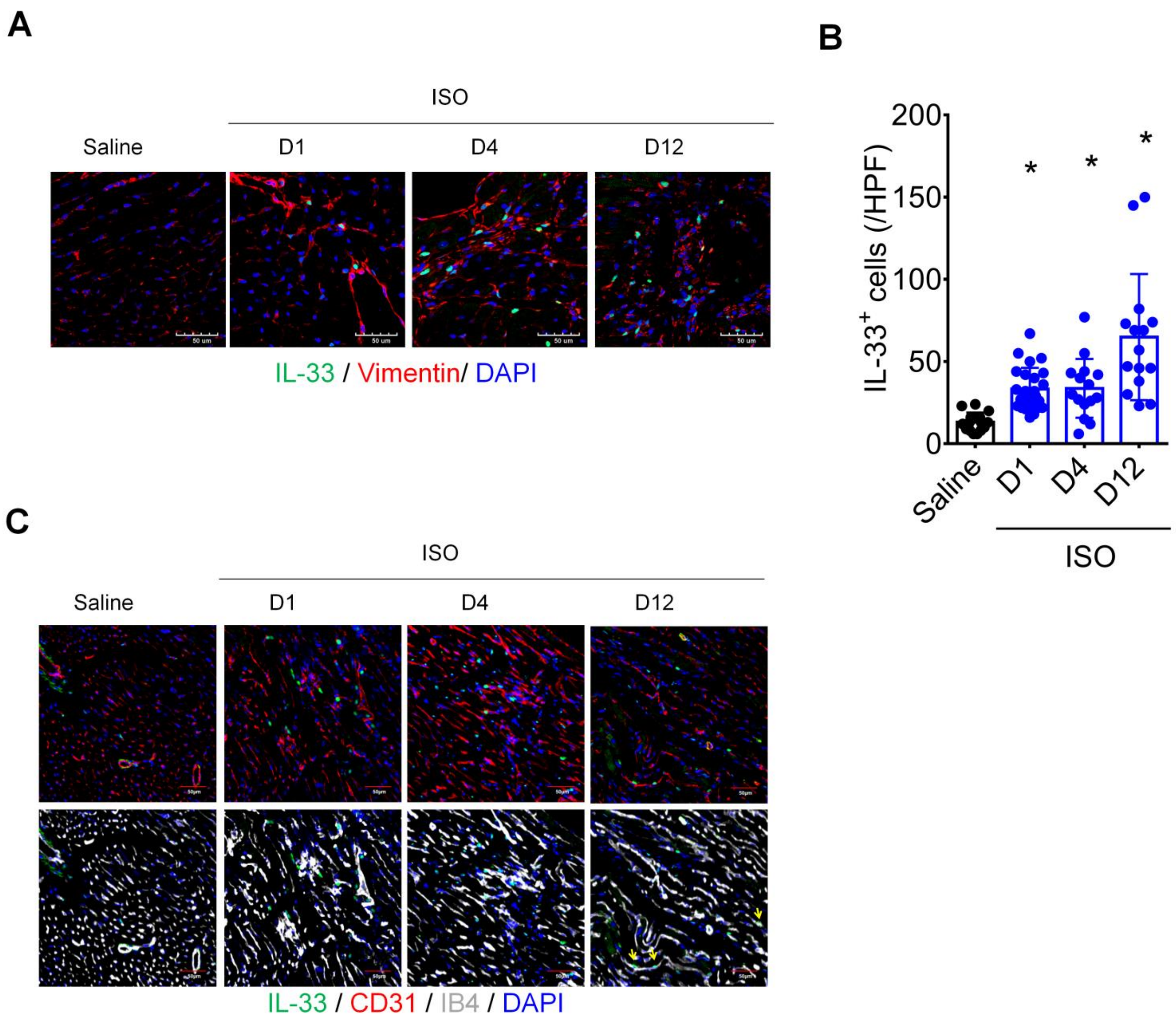

Figure S2. IL-33 expression is upregulated following isoproterenol (ISO)-induced cardiac injury. BALB/cByJ mice (n=3 in each group) were subcutaneously administered with ISO (60 mg/kg) for 3 days. The heart tissues were collected on day 1, 4, and 12 after the last ISO treatment. **(A)** Representative images of immunofluorescence staining for IL-33 (green), Vimentin (red), and nuclei (blue). **(B)** Quantification of IL-33^{+} cells per high-power field (200X magnification). **(C)** Representative images of immunofluorescence staining for IL-33 (green), CD31 (red), isolectin IB4 (IB4) (gray), and nuclei (blue).

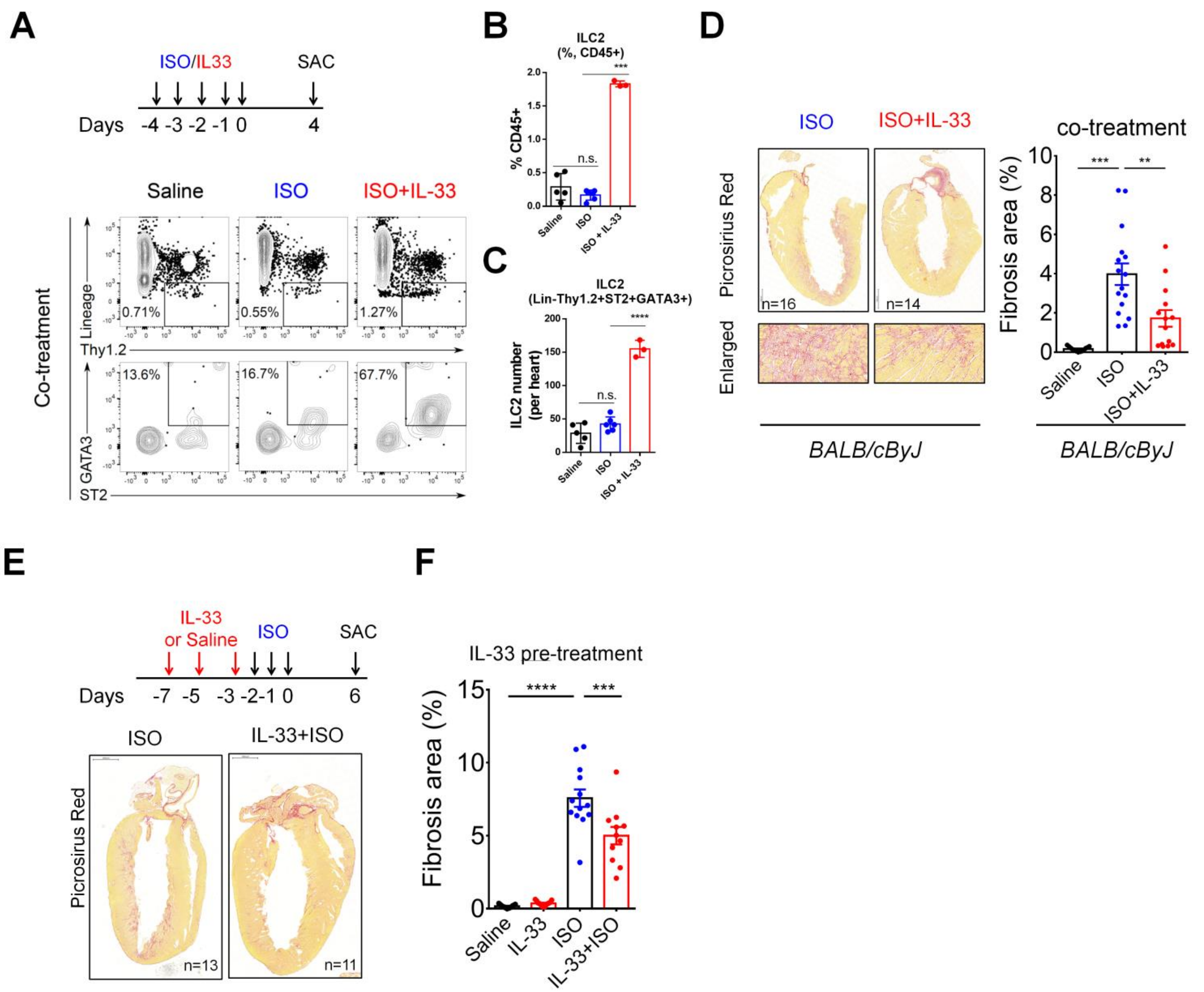

Figure S3. Co-treatment or pre-treatment of IL-33 reduces ISO-induced cardiac fibrosis. (A) BALB/cByJ mice were subcutaneously administered with saline, isoproterenol (ISO), or ISO+ IL-33 for 5 days. On day 4 after the last ISO injection, mice were euthanized (SAC), and cardiac tissues were collected for histological analysis. Flow cytometry analysis of the $CD45^+Lin^-Thy1.2^+GATA3^+$ ILC2 population in the hearts. **(B)** Frequency of ILC2 among $CD45^+$ cells. **(C)** Cell number of ILC2s per heart. **(D)** Picrosirius red staining. Quantification of the cardiac fibrosis area. Scale bar = 100 μm. **(E-F)** BALB/cByJ mice were pre-treated with intraperitoneally administered saline or IL-33 (0.5 μg/mouse) for a total of 3 doses. Then the mice were subcutaneously administered saline or ISO (60 mg kg^{-1} day^{-1}) for 3 days. The cardiac tissues were collected for histological analysis on day 6 after the last ISO injection (SAC). **(E)** Picrosirius red staining for fibrosis area. **(F)** Quantification of cardiac fibrosis area. ***$P < 0.001$, ****$P<0.0001$ by one-way ANOVA followed by the Bonferroni multiple comparison post-hoc test. All values are means ± SD. Each dot indicates a biological replicate.

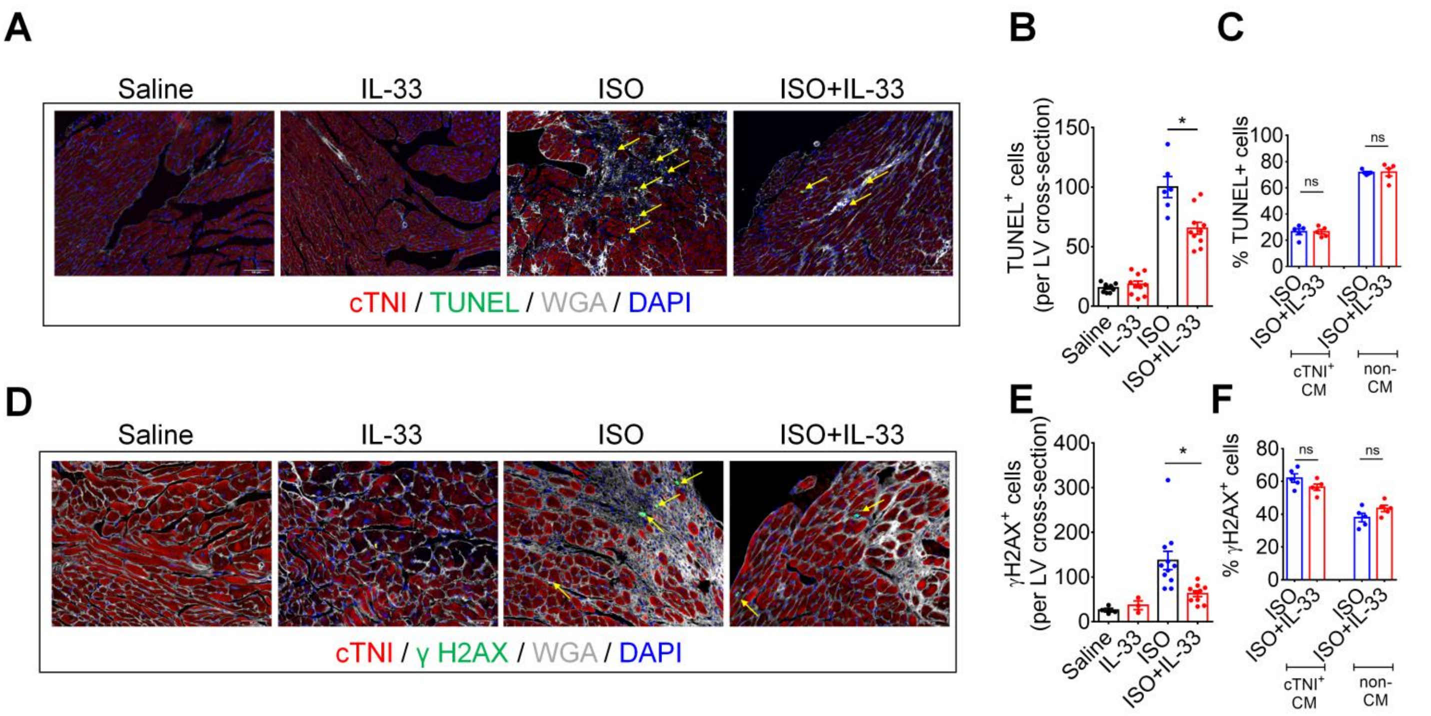

Figure S4. IL-33 treatment ameliorates ISO-induced cell death. (A) Representative images of immunofluorescent staining and **(B)** quantification of TUNEL$^+$ cells in the left ventricle region. Yellow arrowheads indicate respectively labeled cells. Number of positive stained cells in the left ventricle region was quantified in each heart cross-section (8–10 immunofluorescent images at 200X magnification). **(C)** Percentages of cTNI$^+$TUNEL$^+$ CMs and cTNI$^-$TUNEL$^+$ non-CMs among the total TUNEL$^+$ cells. **(D)** Representative images of immunofluorescent staining and **(E)** quantification of γH2AX$^+$ cells in the left ventricle region. Yellow arrowheads indicate respectively labeled cells. Number of positive stained cells in the left ventricle region was quantified in each heart cross-section (8–10 immunofluorescent images at 200X magnification). **(F)** Percentages of cTNI$^+$γH2AX$^+$ CMs and cTNI$^-$γH2AX$^+$ non-CMs among the total γH2AX$^+$ cells. ns, no significance; **P* < 0.05 by one-way ANOVA followed by the Bonferroni multiple comparison post-hoc test. All values are means ± SD. Each dot indicates a biological replicate.

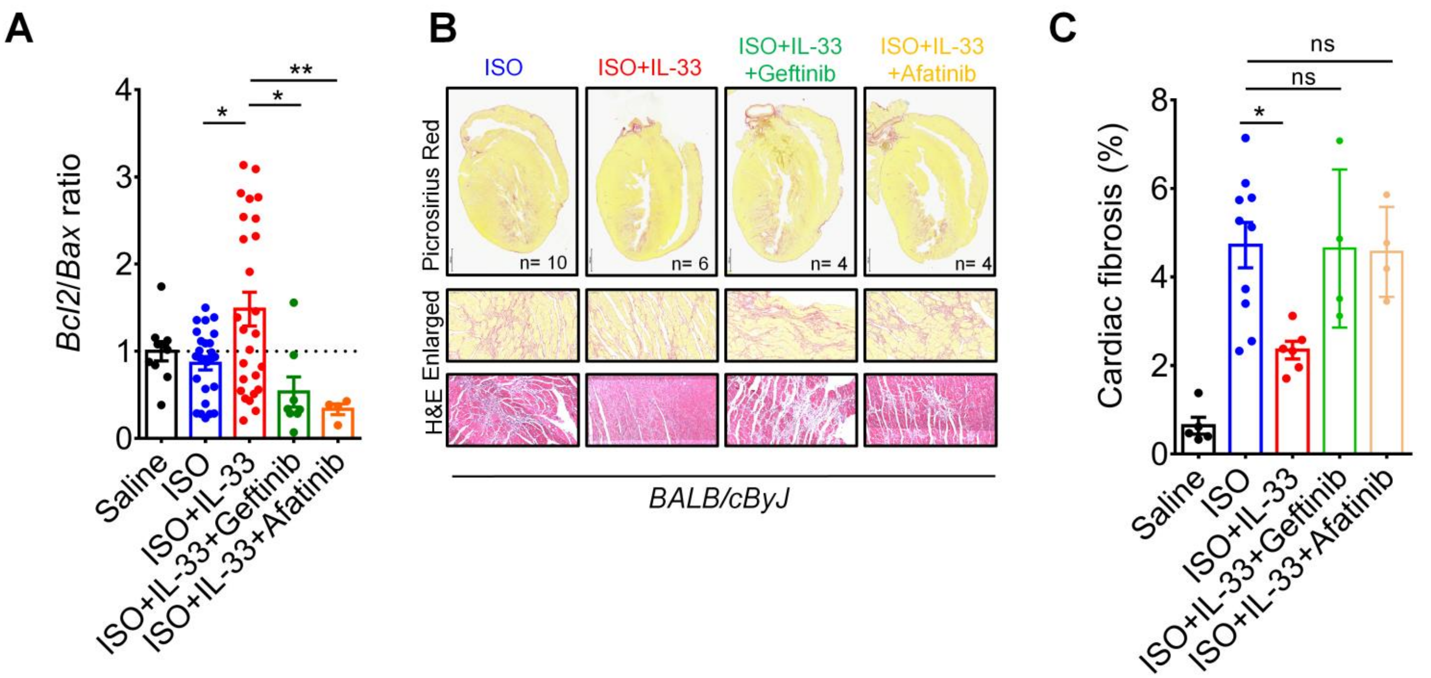

Figure S5. EGFR Signaling is required for optimal function of IL-33 to mitigate cardiac fibrosis. The mice were intraperitoneally administered with Saline, IL-33 (0.5 μg/mouse), IL-33 (0.5 μg) + Geftinib (10 mg/kg), or IL-33 (0.5 μg) + Afatinib (10 mg/kg) on day 3, 5, and 7. **(A)** Ratio of *Bcl2* versus *Bax* gene expression levels in the cardiac tissues. *$P < 0.05$, **$P < 0.01$ by one-way ANOVA followed by the Bonferroni multiple comparison post-hoc test. The fold increase represents relative gene expression compared with saline-treated controls. **(B)** The cardiac tissues were collected for Picrosirius red staining of the fibrotic area. **(C)** Quantification of the fibrosis area in the heart sections. *$P < 0.05$. ns, not significant by one-way ANOVA followed by the Bonferroni multiple comparison post-hoc test. All values are means ± SD. Each dot indicates a biological replicate.

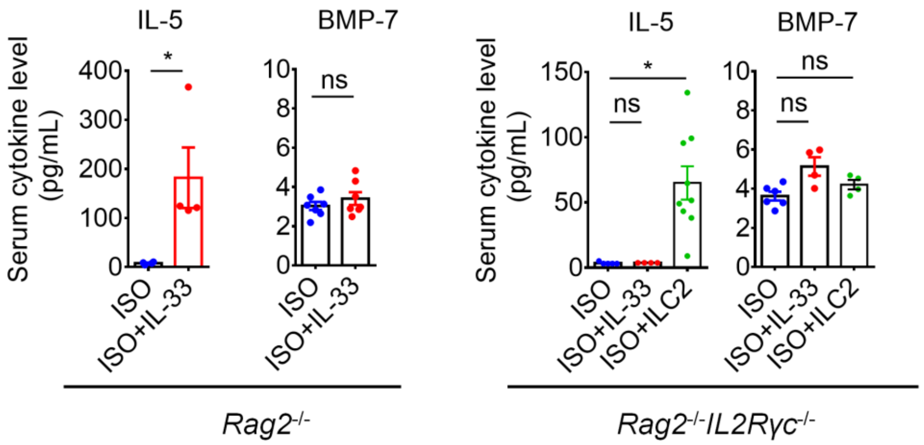

Figure S6. ILC2 contribute to IL-33-induced circulating IL-5. *Rag2*$^{-/-}$ and *Rag2*$^{-/-}$*IL2Rγc*$^{-/-}$ mice were subcutaneously administered with isoproterenol (ISO; 30 mg/kg) for 3 days and intraperitoneally administered with saline or IL-33 (0.5 μg/mouse) on days 3, 5, and 7. The sera were collected on day 10 for ELISA analysis of IL-5 and BMP-7 levels. ns, not significant by one-way ANOVA followed by the Bonferroni multiple comparison post-hoc test.
